# Anatomical Evidence for a Direct Projection from Purkinje Cells in the Mouse Cerebellar Vermis to Medial Parabrachial Nucleus

**DOI:** 10.3389/fncir.2018.00006

**Published:** 2018-02-07

**Authors:** Mitsuhiro Hashimoto, Akihiro Yamanaka, Shigeki Kato, Manabu Tanifuji, Kazuto Kobayashi, Hiroyuki Yaginuma

**Affiliations:** ^1^Department of Neuroanatomy and Embryology, Fukushima Medical University Graduate School of Medicine, Fukushima, Japan; ^2^Brain Interdisciplinary Research Division, Research Institute for Science and Technology, Tokyo University of Science, Noda-shi, Japan; ^3^Department of Neuroscience II, Research Institute of Environmental Medicine, Nagoya University, Nagoya-shi, Japan; ^4^Laboratory for Integrative Neural Systems, RIKEN Brain Science Institute, Saitama, Japan; ^5^Department of Molecular Genetics, Institute of Biomedical Sciences, Fukushima Medical University Graduate School of Medicine, Fukushima, Japan; ^6^Department of Life Science and Medical Bio-Science, Faculty of Science and Engineering, Waseda University, Tokyo, Japan; ^7^Department of Complexity Science and Engineering, Graduate School of Frontier Sciences, University of Tokyo, Kashiwa, Japan

**Keywords:** cerebellum circuits, medial parabrachial nucleus, aden-associated virus, retrograde tracing, anterograde tracing, cerebellar vermis

## Abstract

Cerebellar malformations cause changes to the sleep-wake cycle, resulting in sleep disturbance. However, it is unclear how the cerebellum contributes to the sleep-wake cycle. To examine the neural connections between the cerebellum and the nuclei involved in the sleep-wake cycle, we investigated the axonal projections of Purkinje cells in the mouse posterior vermis by using an adeno-associated virus (AAV) vector (serotype rh10) as an anterograde tracer. When an AAV vector expressing humanized renilla green fluorescent protein was injected into the cerebellar lobule IX, hrGFP and synaptophysin double-positive axonal terminals were observed in the region of medial parabrachial nucleus (MPB). The MPB is involved in the phase transition from rapid eye movement (REM) sleep to Non-REM sleep and *vice versa*, and the cardiovascular and respiratory responses. The hrGFP-positive axons from lobule IX went through the ventral spinocerebellar tract and finally reached the MPB. By contrast, when the AAV vector was injected into cerebellar lobule VI, no hrGFP-positive axons were observed in the MPB. To examine neurons projecting to the MPB, we unilaterally injected Fast Blue and AAV vector (retrograde serotype, rAAV2-retro) as retrograde tracers into the MPB. The cerebellar Purkinje cells in lobules VIII–X on the ipsilateral side of the Fast Blue-injected MPB were retrogradely labeled by Fast Blue and AAV vector (retrograde serotype), but no retrograde-labeled Purkinje cells were observed in lobules VI–VII and the cerebellar hemispheres. These results indicated that Purkinje cells in lobules VIII–X directly project their axons to the ipsilateral MPB but not lobules VI–VII. The direct connection between lobules VIII–X and the MPB suggests that the cerebellum participates in the neural network controlling the sleep-wake cycle, and cardiovascular and respiratory responses, by modulating the physiological function of the MPB.

## Introduction

The parabrachial nucleus is located in the surrounding region of the superior cerebellar peduncle (scp) and is divided into the dorsolateral region, the lateral parabrachial nucleus (LPB) and the ventromedial region, the medial parabrachial nucleus (MPB). The LPB and MPB function as relay nuclei of neural information. The LPB is involved in sodium intake (Geerling and Loewy, 2008; Geerling et al., 2011), respiration (Chamberlin, 2004; Dutschmann and Dick, 2012; Miller et al., 2012; Nisimaru et al., 2013), pain response (Hermanson and Blomqvist, 1996; Richard et al., 2005), thermosensation (for a review see Morrison and Nakamura, 2011), and appetite suppression (Dipatrizio and Simansky, 2008; Wu et al., 2009, 2012; Carter et al., 2013). The MPB is involved in sleep-stage transition (Fuller et al., 2011; Anaclet et al., 2012), especially switching from rapid eye movement (REM) sleep to Non-REM sleep and *vice versa* (Hayashi et al., 2015), cardiovascular and respiratory responses (Nisimaru, 2004; Song et al., 2006), and taste (Rosen et al., 2011; Tokita and Boughter, 2012).

Human patients with cerebellar diseases suffer from sleep disturbance (insomnia, excessive daytime sleepiness, REM behavior disorder, and sleep apnea) (Pedroso et al., 2011; DelRosso and Hoque, 2014; Canto et al., 2017). In addition, cats with lesions of the cerebellar vermis and hemispheres (de Andrés and Reinoso-Suarez, 1979; de Andrés et al., 2011) and cerebellectomized cats (Cunchillos and De Andres, 1982) showed abnormal sleep-wake cycles. In rabbits, Purkinje cells in cerebellar lobule III, the lateral-most region of cerebellar lobule IX, and the flocculus (FL) directly project to the LPB, and are involved in the modulation of cardiovascular and respiratory responses (Supple and Kapp, 1994; Sadakane et al., 2000; Nisimaru et al., 2013). These studies suggest that the cerebellum contributes to regulating the sleep-wake cycle, and the cardiovascular and respiratory responses. Interestingly, the neurons of the LPB and MPB originate in *Atoh1*/*Math1*-positive progenitor cells in the rostral rhombic lip, which is identical to the origin of cerebellar granule cells (Machold and Fishell, 2005; Wang et al., 2005; Rose et al., 2009; Hayashi et al., 2015). Therefore, the cerebellum seems to be closely related to the parabrachial nucleus with respect to neural development, neural circuit formation, and physiological function, but the relationship between the cerebellum and the parabrachial nucleus has been unclear in rodents.

In this study, we used an AAV vector (serotype rh10) expressing hrGFP as an anterograde tracer and Fast Blue and AAV vector (retrograde serotype, rAAV2-retro; Tervo et al., 2016) as retrograde tracers to investigate the neural circuit of the cerebellum. Using the AAV vector (serotype rh10), we found that lobules VIII and IX of mouse cerebellar vermis directly projected their axons to the MPB. Furthermore, retrograde labeling of the MPB indicated that the Purkinje cells of lobules VIII–X directly projected their axons to the ipsilateral MPB. The existence of direct axonal projection from lobules VIII–X to the MPB suggests that the cerebellum contributes to the regulation of the sleep-wake cycle, and of cardiovascular and respiratory responses, by modulating the neural activity of the MPB.

## Materials and methods

### Preparation of an adeno-associated viral vector

AAV-CMV-hrGFP is a recombinant AAV (serotype rh10) which expresses hrGFP; Agilent Technologies, Santa Clara, CA, USA) under the control of human cytomegalovirus (CMV) immediate early enhancer and promoter. hrGFP is a green fluorescent protein derived from *Renilla reniformis* and has a lower cytotoxicity than a green fluorescent protein derived from *Aequorea victoria*. A packaging plasmid of AAV serotype rh10 was purchased from the Penn Vector Core-Gene Therapy Program (University of Pennsylvania, Philadelphia, PA, USA). AAV-CMV-hrGFP was constructed with a pAAV-hrGFP plasmid (Agilent Technologies) and the packaging plasmid of AAV serotype rh10. The packaging, purification, and titration of AAV-CMV-hrGFP was performed as described previously (Inutsuka et al., 2014; Miyamoto et al., 2016).

The AAV vector, AAV2retro-CAG-EGFP was constructed with a AAV-CAGGS-EGFP plasmid that was a gift from Rudolf Jaenisch (Addgene plasmid #22212; Hockemeyer et al., 2009) and a packaging plasmid of rAAV2-retro helper that was a gift from Alla Karpova and David Schaffer (Addgene plasmid #81070; Tervo et al., 2016). AAV2retro-CAG-EGFP expressed enhanced green fluorescent protein (EGFP), which is derived from *A. victoria*, under the control of CAG promoter. The packaging, purification, and titration of AAV2retro-CAG-EGFP was performed as described previously (Kaneda et al., 2011). Briefly, AAV2retro-CAG-EGFP was purified twice by cesium gradient ultracentrifugation and the titer was determined by quantitative PCR for *EGFP*.

### Animals

ICR mice (Slc:ICR; NipponSLC, Sizuoka, Japan) were housed in a controlled environment under a 12-h light:dark cycle. All procedures involving animal preparation were approved by the Fukushima Medical University Animal Committee.

### Injection of AAV vector

The stereotaxic coordinates and nomenclature of each brain region were based on Paxinos and Franklin (2001). A heat-pulled glass micropipette (tip diameter 5 μm) attached to a microinjector (IM-300, Narishige, Tokyo, Japan) was filled with AAV-CMV-hrGFP solution (2 × 10^12^ particles/ml) or AAV2retro-CAG-EGFP solution (2 × 10^12^ particles/ml).

Adult ICR mice were anesthetized by intraperitoneal injection of a mixture of three anesthetic agents [0.3 mg/kg body weight medetomidine hydrochloride (Nippon Zenyaku Kogyo Co., Ltd., Fukushima, Japan), 4.0 mg/kg body weight midazolam (Sandoz, Tokyo, Japan), 5.0 mg/kg body weight butorphanol (Meiji Seika Pharma Co. Ltd., Tokyo, Japan; Kawai et al., 2011)]. After deep anesthesia, the mice were secured on a stereotaxic apparatus (SRS-A, Narishige, Tokyo, Japan). A midline incision was made at the occipital region of the scalp. The skull and the trapezius muscle were exposed. A midline incision was made along the nuchal ligament. The occipital bone and the atlantooccipital membrane were exposed. Cerebellar lobule IX was observed under the posterior edge of the occipital bone through the atlantooccipital membrane. For AAV-CMV-hrGFP injection into the cerebellar lobule IX (*n* = 6, male), a small transversal incision was made at the atlantooccipital membrane for insertion of the glass micropipette. For AAV-CMV-hrGFP injection into the cerebellar lobule VI (*n* = 5, male), a 2-mm-diameter hole was made on the occipital bone at the point 7 mm caudal from bregma with a high-speed drill. For AAV-CMV-hrGFP injection into the cerebellar lobule VIII (*n* = 5, male), a 2-mm-diameter hole was made on the occipital bone at the point 8.5 mm caudal from bregma with a high-speed drill. The injection site of AAV-CMV-hrGFP was the center of the lateral half of the cerebellar lobule. The tip of the glass micropipette was inserted 0.5 mm deep to the surface of the cerebellar lobule. AAV-CMV-hrGFP solution (0.2 μl) was injected bilaterally into the cerebellar lobule through the micropipette. AAV-CMV-hrGFP injection into the MPB (*n* = 4, male) and AAV2retro-CAG-EGFP injection into the MPB (*n* = 6, male) were performed in accordance with the description in the section of Injection of Fast Blue (see below). AAV-CMV-hrGFP solution (0.2 μl) or AAV2retro-CAG-EGFP solution (0.01 μl) was injected into one side of the MPB through the micropipette. After the injection, the skin was sutured. One to two weeks later, the mice were fixed with an intracardiac perfusion of phosphate-buffered saline without calcium and magnesium PBS(–) and 4% paraformaldehyde (PFA) in 0.1 M phosphate buffer (PB) (pH 7.4). The brains were removed and further fixed in the same fixative for 1 h at 4°C. The brains were cryoprotected by serial equilibration in sucrose [10, 15, and 20% (w/v) in PBS(–)] at 4°C, frozen in a Tissue-Tek O.C.T. Compound (Sakura Finetek USA, Inc., Torrance, CA, USA), and sectioned transversely (20 μm thickness) on a cryostat (CM3050S, Leica, Solms, Germany).

### Injection of fast blue

Fast Blue (17740-1, Polyscience, Inc., Warrington, PA, USA) is a fluorescent dye (excitation wavelength 365 nm, emission wavelength 420 nm) most commonly used as a retrograde neuronal tracer. Fast Blue is effectively transported retrogradely over long distances in mouse models (Schofield, 2008). In the present study, Fast Blue was diluted in sterilized water [5% (w/v)]. A heat-pulled glass micropipette (tip diameter 5 μm), which was attached to a microinjector (IM-300, Narishige), was filled with the diluted Fast Blue solution. The micropipette was attached to a micromanipulator (SM-15, Narishige) of the stereotaxic apparatus (SRS-A, Narishige). Adult ICR mice were anesthetized by intraperitoneal injection of a mixture of three anesthetic agents (Kawai et al., 2011). Under deep anesthesia, the mice were secured on a stereotaxic platform (SRS-A, Narishige). The tooth bar was set 2 mm below the point of the ear bar. A midline incision was made in the scalp and the skull was exposed. A 2-mm-diameter hole was made at the point 5.3 mm caudal and 0.9 mm lateral from bregma. Fast Blue solution (0.2 μl) was slowly injected into one side of the MPB (anteroposterior from bregma, −5.3 mm; mediolateral from bregma, 0.9 mm; dorsoventral from bregma, 3.4 mm) through the micropipette (*n* = 10, male). In the case of Fast Blue-injection into cerebellar lobule IX (*n* = 5, male), Fast Blue was injected into cerebellar lobule IX in the same way as the injection of AAV vector. In the case of Fast Blue-injection into medial vestibular nucleus (MVe), a 2-mm-diameter hole was made at the point 6 mm caudal and 0.7 mm lateral from bregma. Fast Blue solution (0.2 μl) was slowly injected into one side of the MVe (anteroposterior from bregma, −6 mm; mediolateral from bregma, 0.7 mm; dorsoventral from bregma, 3.9 mm) through the micropipette (*n* = 4, male). After the injection, the skin was sutured. 1 week later, the mice were fixed with an intracardiac perfusion of PBS(–) and 4% PFA in 0.1 M PB (pH 7.4). The brains were removed and further fixed in the same fixative for 1 h at 4°C. The brains were cryoprotected by serial equilibration in sucrose [10, 15, and 20% (w/v) in PBS(–)] at 4°C, frozen in a Tissue-Tek O.C.T. Compound (Sakura Finetek USA, Inc.), and sectioned transversely (20 μm thickness) on a cryostat (CM3050S, Leica).

### Immunohistochemistry

Tissue sections were soaked with PBS(–) and incubated for 1 h in PBS(–) with 1% skim milk (Wako, Tokyo, Japan) and 0.1% Triton X-100 (blocking solution), followed by incubation with the primary antibody diluted in the blocking solution overnight at 4°C. Sections were washed several times with PBS(–) and incubated with the appropriate fluorescence-conjugated secondary antibody for 2 h. The primary antibodies were as follows: rabbit polyclonal anti-GFP (1:500, #598, Medical and Biological Laboratories, Co., Ltd., Nagoya, Japan), rabbit polyclonal anti-Tyrosine hydroxylase (TH; 1:250, #2792, Cell Signaling Technology Japan, K. K., Tokyo, Japan), mouse monoclonal anti-synaptophysin [SYP (SVP38); 1:200, sc-12737, Santa Cruz Biotechnology, Inc., Dallas, TX, USA], mouse monoclonal anti-vesicular glutamate transporter 2 (VGLUT2; clone 8G9.2, 1:100, MAB5504, Merck Millipore, Darmstadt, Germany, and mouse monoclonal anti-calcitonin gene-related peptide (CGRP; clone 4901, 1:100, sc-57053, Santa Cruz Biotechnology, Inc.). VGLUT2 (Rose et al., 2009; Krenzer et al., 2011) and CGRP (Schwaber et al., 1988; Dobolyi et al., 2005; D'Hanis et al., 2007; Miller et al., 2012) are expressed in the MPB and the ventrolateral region of MPB, respectively. The fluorescence-labeled secondary antibodies were as follows: rhodamine-conjugated goat anti-rabbit IgG (111-025-144, 1:200, Jackson ImmunoResearch Laboratories, Inc., West Grove, PA, USA) and rhodamine-conjugated donkey anti-mouse IgG (715-025-151, 1:200, Jackson ImmunoResearch Laboratories, Inc.). The fluorescence-labeled sections were mounted under coverslips with Fluoromount^MT^ (ID Labs, Inc., London, Canada). Immunoreactivity against GFP was detected by using a goat biotinylated anti-rabbit antibody (1:200, #BA-1000, Vector, Burlingame, CA), an ABC kit (VECTASTAIN Standard Elite, Vector), and DAB (Dojindo Molecular Technologies, Tokyo, Japan). The DAB-stained sections were mounted under coverslips with Permount (Fisher Scientific, Somerville, NJ) using a standard procedure.

### Image acquisition

Optical images of whole cerebellums were obtained with a fluorescent stereomicroscope (MZ16F, Leica) attached to a CCD camera (DP-70, Olympus, Tokyo, Japan). The images of sections were obtained with a fluorescent microscope (BX-50, Olympus) attached to a CCD camera (DP-71, Olympus) and an all-in-one fluorescent microscope (BZ-X700, Keyence Corporation, Osaka, Japan). The image data were manipulated with Photoshop CS5 (Adobe Systems, San Jose, CA, USA) to adjust the color balance and the degree of brightness. Images of fluorescence were converted to black and white with Photoshop CS5 (Adobe Systems). Some of the monochrome images were inverted to black-and-white by Photoshop CS5 (Adobe Systems). Some images were traced in Illustrator CS5 (Adobe Systems). The confocal image data (immunohistochemistry for SYP and for VGLUT2: image size, 800 × 800 pixels; resolution, 0.265 μm/pixel; immunohistochemistry for CGRP: image size, 800 × 800 pixels; resolution, 0.106 μm/pixel) were acquired with a confocal fluorescence microscope (FV-1000, Olympus) and stored on a computer by using FLUOVIEW software (Olympus). The confocal image data were reconstructed into a stacked image by ImageJ software (Schneider et al., 2012). Some stacked images were converted into a flattened, single image using the Z projection-Maximum Intensity command of ImageJ software. Some stacked images were reconstructed into 3D images by using the 3D projection-Brightest Point command of ImageJ software, and the 3D images were saved as movies.

## Results

### Anterograde labeling of cerebellar lobule IX by AAV-CMV-hrGFP

**Figure 1:**
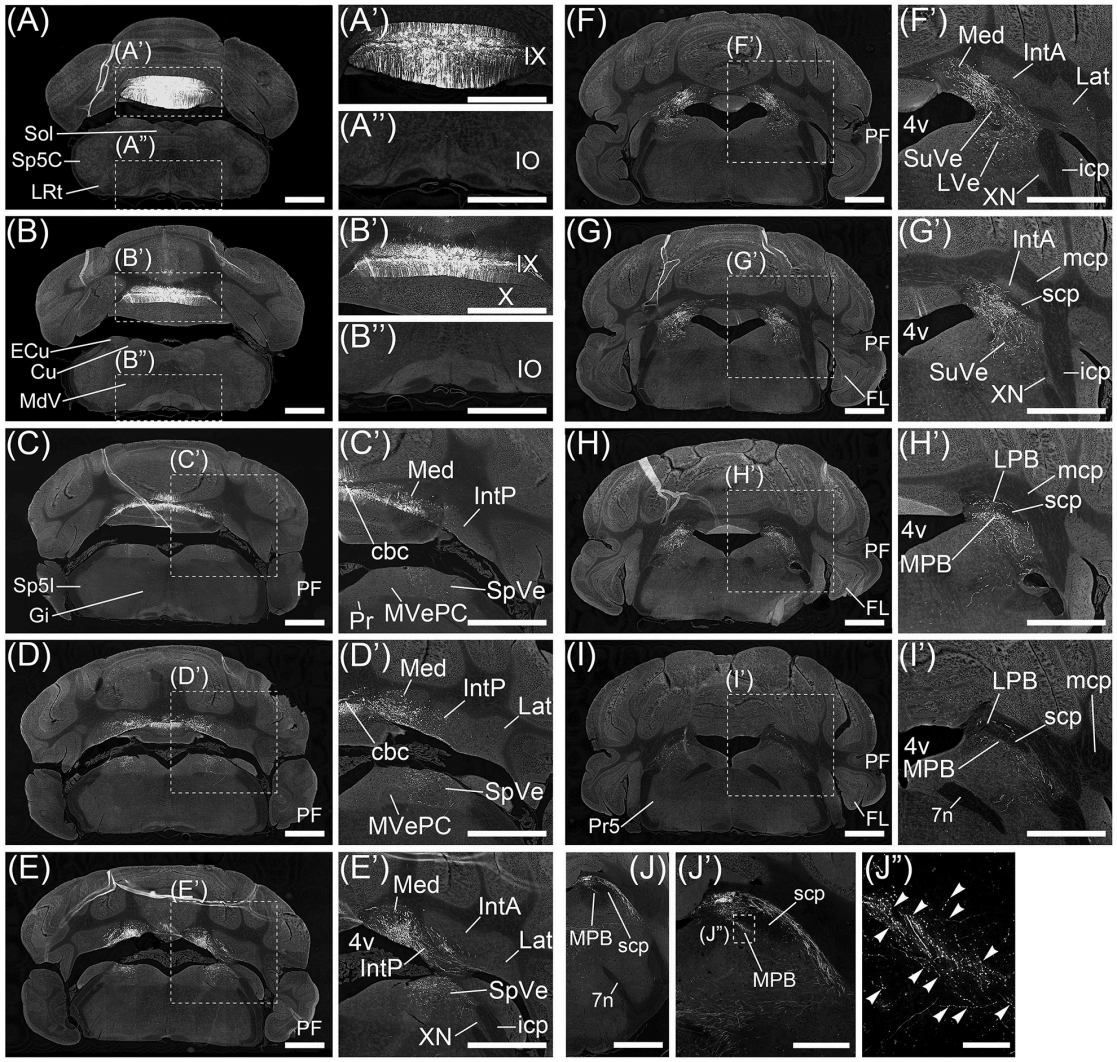
Regions projected from cerebellar lobule IX. Axons from cerebellar lobule IX were anterogradely labeled by AAV-CMV-hrGFP. **(A–I)** Series of transversal sections from posterior to anterior cerebellum. Fluorescence of hrGFP on each section was imaged. **(A'–I', A”, B”)** The magnified images of quadrilateral regions in each section. Neurons in lobule IX are selectively and densely labeled by hrGFP (**A', B'**). Inferior olive (IO) neurons are not retrogradely labeled by AAV-CMV-hrGFP **(A”, B”)**. hrGFP-positive neurons in cerebellar lobule IX project their axons to medial (fastigial) cerebellar nucleus (Med; **C'–F'**), posterior interposed cerebellar nucleus (IntP; **D', E'**), spinal vestibular nucleus (SpVe; **C'–E'**), superior vestibular nucleus (SuVe; **F', G'**), lateral vestibular nucleus (LVe; **F'**), lateral parabrachial nucleus (LPB; **G'–I'**), and medial parabrachial nucleus (MPB; **G'–I'**). **(J–J”)** Magnified images of MPB. **(J')** The magnified image of the region of MPB and scp in **(J)**. **(J”)** The magnified image of a quadrilateral region in **(J')**. Arrowheads indicate hrGFP-positive axonal varicosities in MPB. 4v, fourth ventricle; IX, cerebellar lobule IX; X, cerebellar lobule X; cbc, cerebellar commissure; Cu, cuneate nucleus; ECu, external cuneate nucleus; FL, flocculus; Gi, gigantocellular reticular nucleus; MVePC, parvocellular part of medial vestibular nucleus; IntA, anterior interposed cerebellar nucleus; icp, inferior cerebellar peduncle; Lat, lateral (dentate) cerebellar nucleus; LRt, lateral reticular nucleus; mcp, medial cerebellar peduncle; MdV, ventral part of medullary reticular nucleus; PCGS, paracochlear glial substance; PF, paraflocculus; Pr, prepositus nucleus; Pr5, principal sensory trigeminal nucleus; scp, superior cerebellar peduncle; Sol, nucleus of the solitary tract; Sp5C, caudal part of spinal trigeminal nucleus; SP5I, interpolar part of spinal trigeminal nucleus; XN, X nucleus. Scale bar, 1 mm in **(A–J)**, **(A'–I'**, **A”**, **B”)**; 500 μm in **(J')**; 50 μm in **(J”)**.

AAV-CMV-hrGFP is a recombinant AAV (serotype rh10) that expresses hrGFP under the control of a human CMV promoter. AAV-CMV-hrGFP was injected into cerebellar lobule IX of adult mice (*n* = 6, male). The fluorescence of hrGFP on a series of transversal sections of AAV-CMV-hrGFP-injected brains was imaged (Figure 1). In the cerebellum, local injection of AAV-CMV-hrGFP labeled only lobule IX with hrGFP (Figures 1A,B,A',B'). The other cerebellar lobules, hemispheres, FL, and paraflocculus (PF) were negative for hrGFP (Figures 1A–I). The linear-shaped structures labeled by hrGFF in lobule IX were dendrites of Purkinje cells and processes of Bergmann glial cells (Figures 1A',B'). Cerebellar interneurons in the molecular layer and granular cell layer were labeled by hrGFP (data not shown). Parallel fibers of cerebellar granular cells were also labeled by hrGFP, but the number of hrGFP-positive parallel fibers was small (data not shown). The infectivity of AAV vector (serotype rh10) to cerebellar granule cells seems to be low compared with Purkinje cells, Bergmann glial cells, and cerebellar interneurons.

No neurons in inferior olive (IO) and pontine nucleus (Pn), which send axonal fibers to the cerebellar cortex as a precerebellar nucleus, expressed hrGFP (Figures 1A”,B” and data not shown). The other precerebellar nuclei (Fu et al., 2011), which are lateral reticular nucleus (LRt, Figure 1A), nucleus of the solitary tract (Sol, Figure 1A), caudal part of spinal trigeminal nucleus (Sp5C, Figure 1A), cuneate nucleus (Cu, Figure 1B), external cuneate nucleus (ECu, Figure 1B), ventral part of medullary reticular nucleus (MdV, Figure 1B), gigantocellular reticular nucleus (Gi, Figure 1C), interpolar part of spinal trigeminal nucleus (Sp5I, Figure 1C), parvocellular part of medial vestibular nucleus (MVePC, Figure 1C'), prepositus nucleus (Pr, Figure 1C'), X nucleus (XN, Figure 1E'), principal sensory trigeminal nucleus (Pr5, Figure 1I), and parvicellular part of motor trigeminal nucleus (data not shown), were negative for hrGFP. The result indicated that AAV-CMV-hrGFP (serotype rh10) had the ability of anterograde labeling but not retrograde labeling.

**Figure 2:**
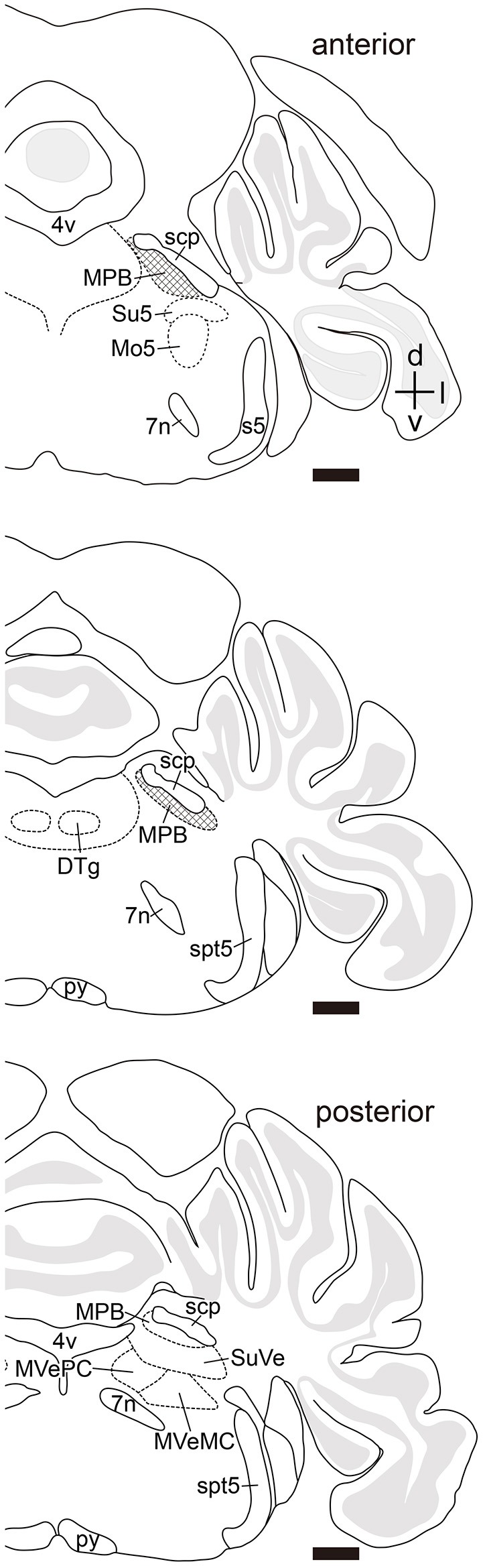
Definition of a region of interest. Schematic drawings indicate serial transverse sections at intervals of 200 μm. The diamond pattern area is defined as a region of interest (ROI). ROI is located in the ventral side of scp and identical to anterior region of the MPB. The posterior region of MPB that is adjacent to the vestibular nuclei was eliminated from the ROI (lower panel). 4v, fourth ventricle; 7n, root of facial nucleus; d, dorsal; DTg, dorsal tegmental nucleus; g7, genu of facial nucleus; l, lateral; Mo5, motor trigeminal nucleus; MVeMC, magnocellular part of medial vestibular nucleus; py, pyramidal tract; s5, sensory root of trigeminal nucleus; spt5, spinal trigeminal tract; Su5, supratrigeminal nucleus; v, ventral. Scale bar, 500 μm.

Many axonal fibers projecting from lobule IX were labeled by hrGFP. The hrGFP-positive axonal fibers were observed in the vicinity of cerebellar commissure (cbc; Figures 1C',D') and in the medial (fastigial) cerebellar nucleus (Med; Figures 1C'–F'), posterior interposed cerebellar nucleus (IntP; Figures 1D',E'), superior vestibular nucleus (SuVe; Figures 1F',G'), lateral vestibular nucleus (LVe; Figure 1F'), spinal vestibular nucleus (SpVe; Figures 1C'–E'), MPB (Figures 1H'–I'), and (LPB; Figures 1H',I'). No hrGFP-positive axonal fibers were observed in the lateral (dentate) cerebellar nucleus (Lat; Figures 1D'–F'), anterior interposed cerebellar nucleus (IntA; Figures 1E'–G'), inferior cerebellar peduncle (icp; Figures 1E',F'), medial cerebellar peduncle (mcp; Figures 1G'–I'), and (scp; Figures 1G'–I', 2). The efferent fibers from lobule IX seemed to run through cbc (Figures 1C',D'), enter into Med (Figures 1C'–F'), IntP (Figures 1D',E'), and expand to SpVe (Figures 1C'–E'), LVe (Figure 1F'), SuVe (Figures 1F',G'), LPB (Figures 1H',I'), and MPB (Figures 1H',I',J,J'). Axonal varicosities, which were likely presynaptic structures, were observed in MPB, Med, IntP, SuVe, LVe, and SpVe (MPB, Figure 1J”, arrowheads; the others, data not shown). The present result was supported by a previous study indicating that neurons in rat lobule IX project their axons to Med, IntP, and vestibular nuclei (LVe, MVe, SpVe, and SuVe,) (Kotchabhakdi and Walberg, 1978; Balaban, 1984; Bernard, 1987). However, to date, there has been no report indicating axonal connection between lobule IX and MPB. We defined a region of interest (ROI) on the MPB (Figure 2, diamond pattern region). The ROI was located in the ventromedial region of scp, identical to the anterior region of MPB, and far from vestibular nuclei [MVePC, magnocellular part of medial vestibular nucleus (MVeMC) and SuVe]. The posterior region of MPB was eliminated from the ROI because it is adjacent to the vestibular nuclei (Figure 2, lower panel). In the ROI on MPB, we observed many hrGFP-positive axonal varicosities (Figures 1J–J”). We examined the axonal connectivity between the cerebellar vermis and the ROI on MPB.

**Figure 3:**
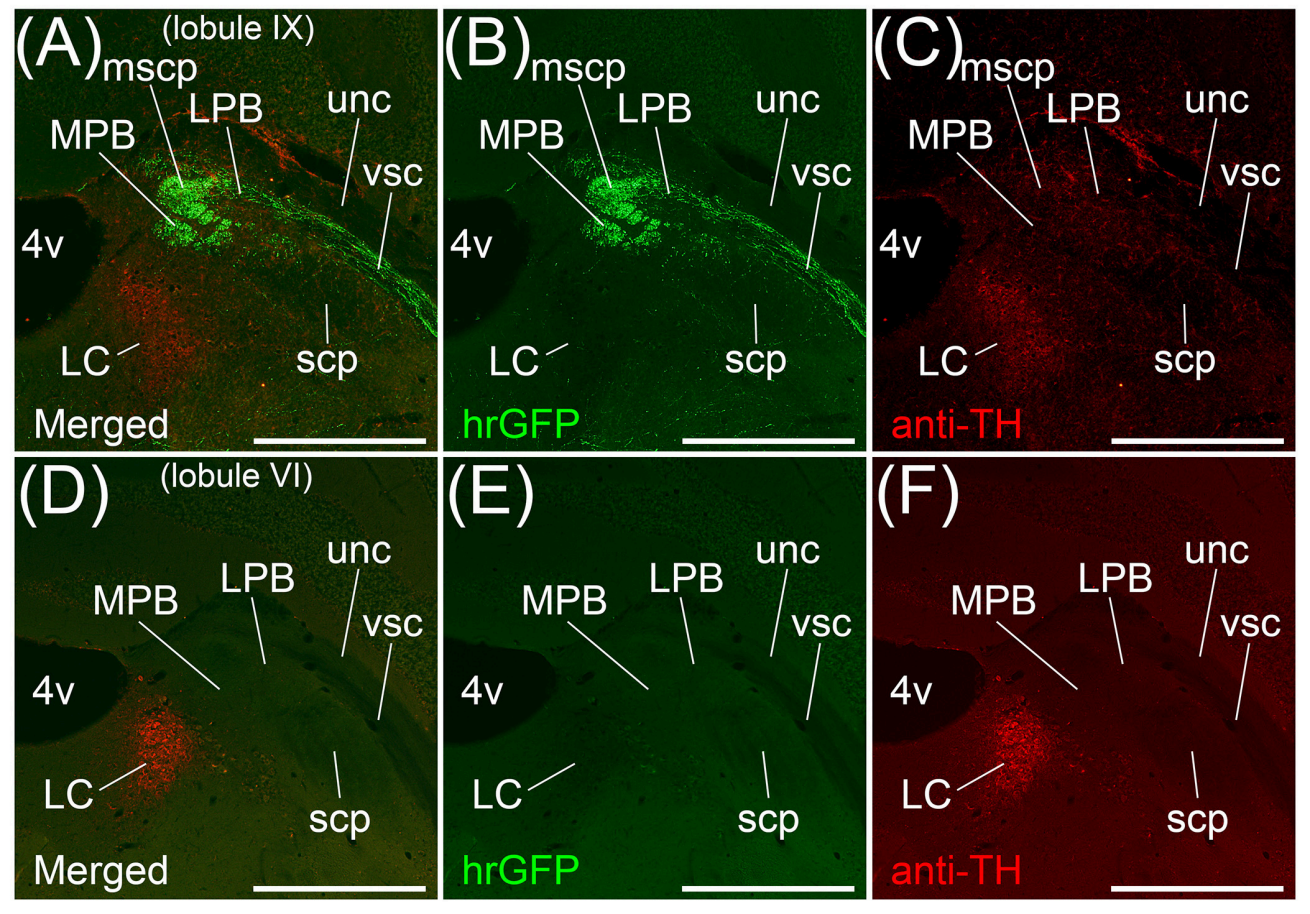
MPB projected from lobule IX but not from lobule VI. **(A–C)** Anterograde labeling from cerebellar lobule IX by AAV-CMV-hrGFP. **(D–E)** Anterograde labeling from cerebellar lobule VI by AAV-CMV-hrGFP (**B,E**). The transversal sections of each labeled brain were immunostained with anti-TH antibody, which is a marker for locus coeruleus (LC) (**C**,**F**). Merged images are indicated in **(A)** and **(D)**. **(A**,**B)** In the case of cerebellar lobule IX labeling, hrGFP-positive axons are abundant in LPB, MPB, the medial-most region of superior cerebellar peduncle (mscp), and ventral spinocerebellar tract (vsc). There are no hrGFP-positive axons in TH-positive LC, scp excluding mscp, and uncinate fasciculus of cerebellum (unc). **(E)** In the case of cerebellar lobule VI labeling, hrGFP-positive axonal terminals are absent in LPB, MPB, and vsc. Scale bar, 500 μm.

Transversal sections of the brains injected with AAV-CMV-hrGFP into lobule IX were immunostained with anti-Tyrosine hydroxylase (TH) antibody, which is a marker for locus coeruleus (LC), to clarify the location of LC (Figures 3A–C). hrGFP-positive axons were observed in LPB, the ROI on MPB, the medial-most region of scp (mscp), and vsc but not in TH-positive LC, uncinate fasciculus of cerebellum (unc), and scp excluding mscp (Figures 3A–C). To examine whether a cerebellar lobule other than lobule IX projected to the ROI on MPB, we injected AAV-CMV-hrGFP into lobule VI (*n* = 5, male). Transversal sections of the brains injected with AAV-CMV-hrGFP into lobule VI were immunostained with anti-TH antibody (Figures 3D–F). In contrast to AAV-CMV-hrGFP injection into lobule IX (Figure 3B), when AAV-CMV-hrGFP was injected into lobule VI, no hrGFP-positive signals were observed in the LPB and the ROI on MPB (Figure 3E). However, hrGFP-positive axons from lobule VI were observed in the Med (data not shown). It is well known that Purkinje cells in the cerebellar vermis generally project their axons to the Med (Steward, 2000; Martin, 2012; Sengul and Watson, 2012). The present results indicated that lobule VI does not project axons to the ROI on MPB.

The main routes of efferent fibers of the cerebellum are the icp, unc, and scp (Steward, 2000; Martin, 2012). Purkinje cells in the FL, PF, and lobule X project their axons to the vestibular nuclei through the icp. Neurons in the Med project their axons to the vestibular nuclei and the spinal cord through the unc and icp. Neurons in the interposed cerebellar nuclei and Lat project their axons to the contralateral thalamus, red nucleus, and spinal cord through the scp. However, efferent fibers from lobule IX to the MPB ran through the vsc (Figures 3A,B) but not the icp (Figures 1E',F'), unc (Figures 3A,B), and scp (Figures 1G'–I', 3A,B). Therefore, the axonal pathway between lobules VIII–X and the MPB differed from the general routes of cerebellar efferent fibers.

**Figure 4:**
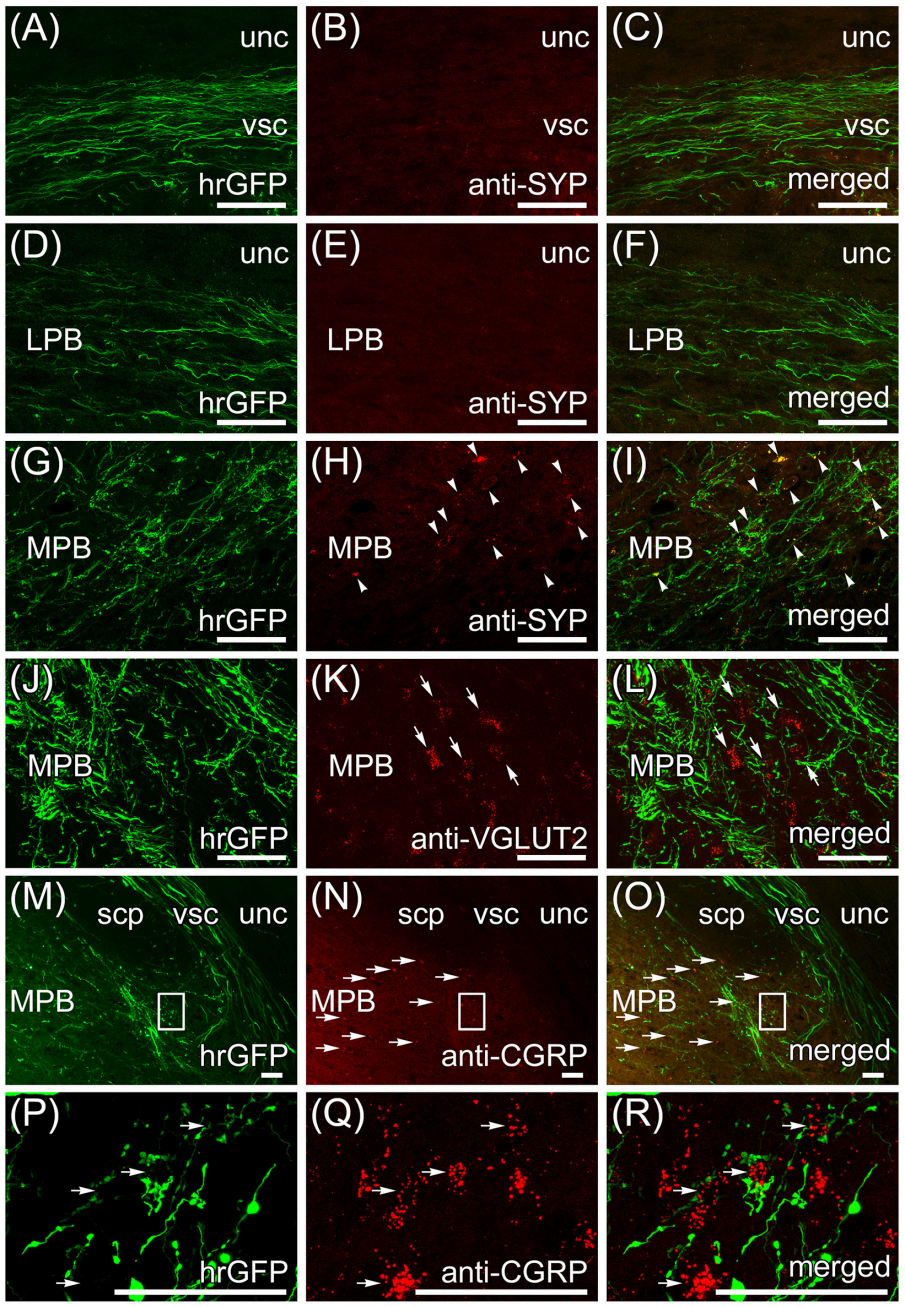
Immunohistochemistry with anti-synaptophysin (SYP) and anti-vesicular glutamate transporter 2 (VGLUT2) antibodies. The transversal sections of brain that are anterogradely labeled by the AAV-CMV-hrGFP injection into lobule IX are immunostained with anti-SYP **(A–I)**, anti-VGLUT2 **(J–L)**, and anti-CGRP **(M–R)** antibodies. Z-stacked confocal images of hrGFP-fluorescence **(A,D,G,J,P)**, z-stacked confocal images of immunostaining **(B,E,H,K,Q)**, and merged images of the hrGFP-fluorescence and the immunostaining **(C,F,I,L,R)** are indicated. **(A–C)** vsc immunostained with anti-SYP antibody. The hrGFP-positive axonal fibers in vsc are negative for SYP. **(D–F)** LPB immunostained with anti-SYP antibody. The hrGFP-positive axonal fibers in LPB are negative for SYP. **(G–I)** MPB immunostained with anti-SYP antibody. The hrGFP-positive axonal terminals in MPB are positive for SYP (arrows). **(J–L)** MPB immunostained with anti-VGLUT2 antibody. Axonal terminals labeled by hrGFP are observed around VGLUT2-positive MPB neurons (arrows). **(M–O)** Immunostaining with anti-CGRP antibody. The hrGFP-positive axonal fibers **(M)** and the CGRP-positive cells [horizontal arrows in **(N,O)**] are observed in ventrolateral region of MPB. **(P–R)** Z-stacked confocal images of the rectangular regions in **(M–O)**. The images rotated counterclockwise by 90 degrees are shown. Axonal terminals labeled by hrGFP are observed around CGRP-positive MPB neurons (horizontal arrows). Scale bar, 50 μm.

Transversal sections of the brain that are anterogradely labeled by the AAV-CMV-hrGFP injection into lobule IX were immunostained with anti-synaptophysin (SYP) antibody, which is a presynaptic marker, and imaged with a confocal microscopy (Figures 4A–I). Many hrGFP-positive axonal fibers were observed in the vsc (Figure 4A) and LPB (Figure 4D), but there were no SYP-immunopositive signals in the vsc (Figure 4B) and LPB (Figure 4E). These results indicated that the hrGFP-positive axonal fibers did not form synapses in the region of the vsc and LPB, and thus, the hrGFP-positive axonal fibers in the vsc and LPB were passing fibers. In the ROI on MPB, many hrGFP-positive axonal fibers were observed (Figure 4G), and furthermore, many hrGFP-positive axonal terminals were positive for SYP (Figures 4H,I, arrowheads). In addition, hrGFP and SYP double-positive signals were observed on the surface of cells in the ROI on MPB (Supplementary Movie 1). These results indicated that hrGFP-positive axonal fibers form synapses on neurons within the ROI on MPB. Furthermore, transversal sections of the brain that are anterogradely labeled by the AAV-CMV-hrGFP injection into lobule IX were immunostained with anti-VGLUT2 and anti-CGRP antibodies, which are markers for MPB neurons (Schwaber et al., 1988; Dobolyi et al., 2005; D'Hanis et al., 2007; Rose et al., 2009; Krenzer et al., 2011; Miller et al., 2012). The immunostained sections were imaged with a confocal fluorescence microscope (anti-VGLUT2, Figures 4J–L; anti-CGRP, Figures 4P–R) and a fluorescence microscope (anti-CGRP, Figures 4M–O). Most of cells within the ROI on MPB were positive for VGLUT2 (Figures 4K,L, arrows) and cells in the ventrolateral region of the ROI on MPB were positive for CGRP (Figures 4N,O, horizontal arrows). In addition, hrGFP-positive axonal terminals were observed on the surface of VGLUT2-positive cells (Figure 4L, arrows; Supplementary Movie 2) and CGRP-positive cells (Figure 4R, horizontal arrows; Supplementary Movie 3). These results indicated that the axonal fibers from cerebellar lobule IX ran along the vsc, passed through the LPB, and finally formed synaptic connection with neurons in the MPB. Taking all the above results into account, we found that the projection from lobule IX to the MPB was a novel neuronal circuit in the cerebellum. Therefore, we further investigated the neuronal connection between lobule IX and the MPB.

### Retrograde labeling from MPB by fast blue

**Figure 5:**
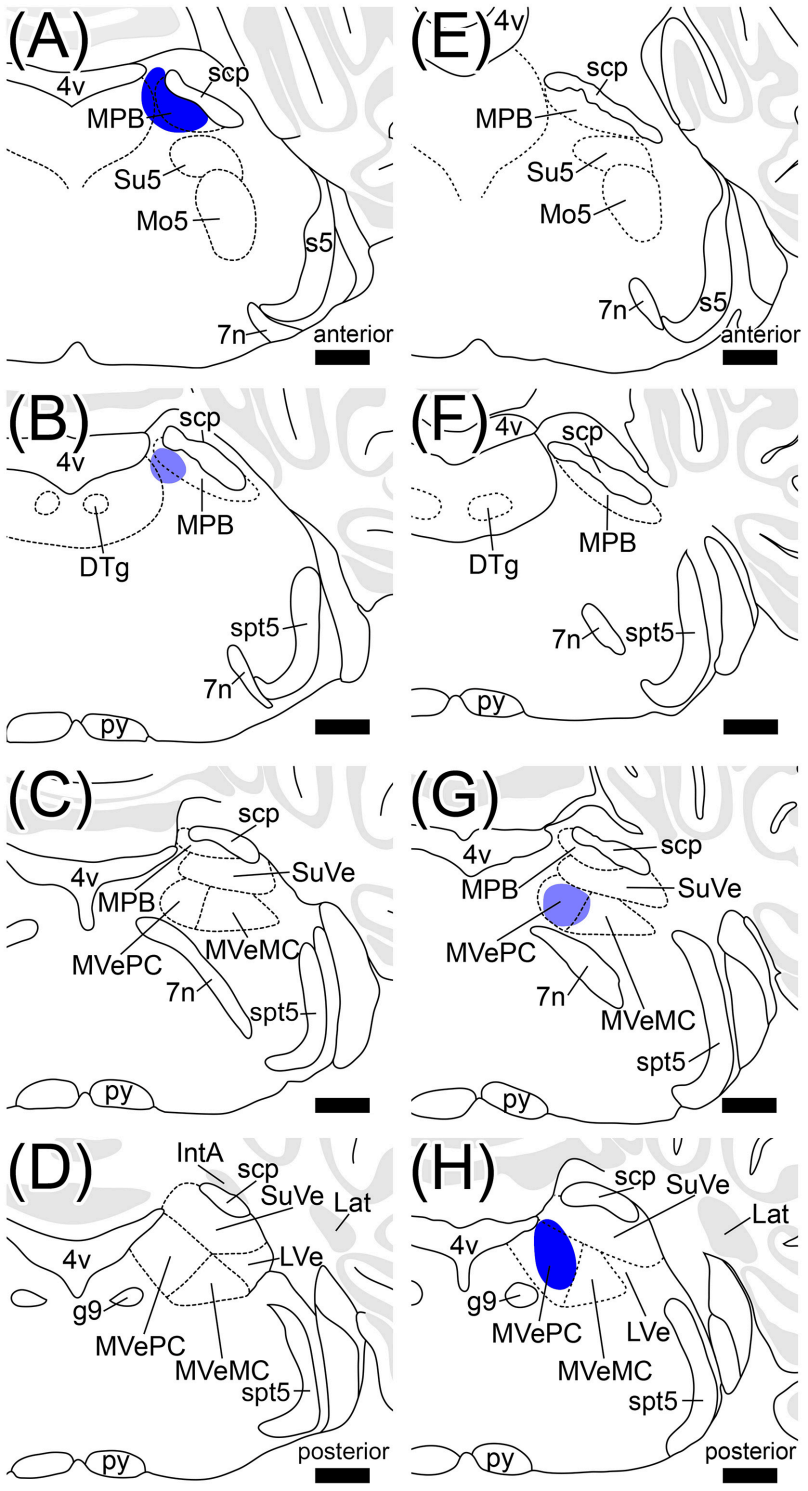
Injection sites of Fast Blue into MPB and MVe. **(A–D)** The Fast Blue-injection site into MPB. **(E–H)** The Fast Blue-injection site into MVe. Schematic drawings **(A–D, E–H)** indicate serial sections at intervals of 200 μm. **(A)** is identical to Figure 6A. **(G)** and **(H)** are identical to Figures 8A,B respectively. The Fast Blue injection sites are shown in blue regions. The intensity of Fast Blue-labeling of **(B,G)** is weaker than it of **(A,H)**. The Fast Blue-injection site to MPB does not overlap with the Fast Blue-injection site to MVe. 7n, root of facial nucleus; DTg, dorsal tegmental nucleus; g7, genu of facial nucleus; Mo5, motor trigeminal nucleus; py, pyramidal tract; s5, sensory root of trigeminal nucleus; spt5, spinal trigeminal tract; Su5, supratrigeminal nucleus. Scale bar, 500 μm.

**Figure 6:**
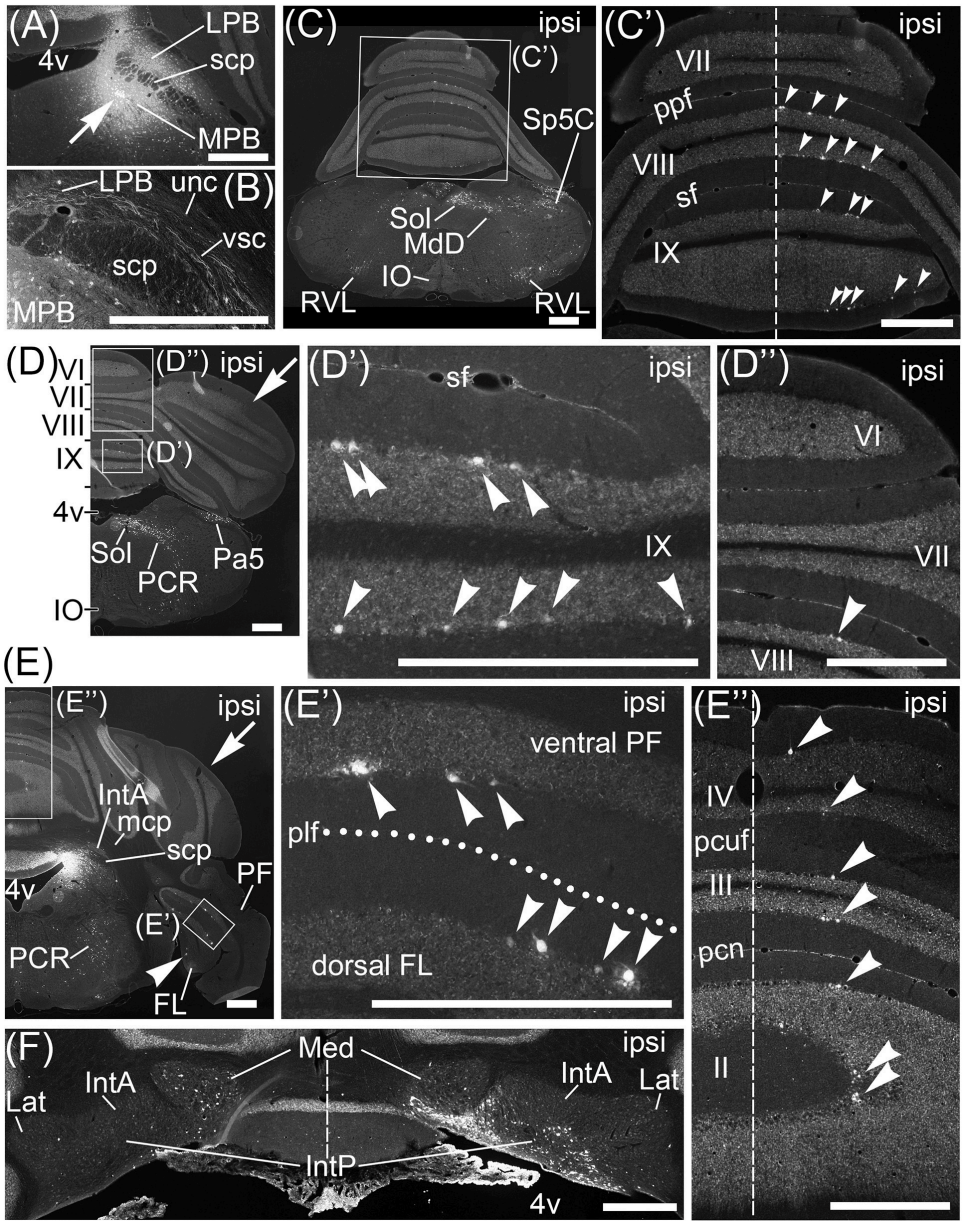
Projection neurons to MPB. Neurons projecting their axons to MPB were retrogradely labeled by Fast Blue. **(A)** Local injection of Fast Blue to MPB. The injection point is indicated by an arrow. **(B)** Magnified image of region including LPB, scp, and vsc. Fas Blue-labeled axons pass through LBP and along vsc. There are no Fast Blue-labeled axons in scp and unc. **(C–E)** Series of transversal sections from posterior to anterior cerebellum. (**C'–E'**, **D”**, **E”**) The magnified images of quadrilateral regions in each section. Arrowheads indicate Purkinje cells labeled retrogradely from MPB by Fast Blue. Fast Blue-labeled Purkinje cells are observed on the ipsilateral side of lobules VIII–IX (**C'**, **D'**, **D”**), lobules II–IV (**E”**), ventral PF (**E**, **E'**), and FL (**E**, **E'**). No Purkinje cells in lobules VI and VII are labeled by Fast Blue (**C'**, **D”**). Dashed lines in (**C')**, **(E”)**, and **(F)** indicate the midline. Dotted line in **(E')** indicates posterolateral fissure (plf). **(F)** Magnified image of bilateral cerebellar nuclei. Ipsilateral IntP is labeled by Fast Blue. By contrast, neurons in contralateral Med are more labeled than in ipsilateral Med. II–IX, numbers of cerebellar lobules; ipsi, ipsilateral; MdD, dorsal part of medullary reticular nucleus; Pa5, paratrigeminal nucleus; pcn, precentral fissure; PCR, parvocellular reticular nucleus; pcuf, preculminate fissure; ppf, prepyramidal fissure; RVL, rostral ventrolateral reticular nucleus; sf, secondary fissure. Scale bar, 500 μm.

To examine brain regions projecting to the MPB, we locally injected Fast Blue, which is a typical retrograde tracer (Schofield, 2008), into the unilateral MPB (*n* = 10, male; Figure 6). The images of Fast Blue-fluorescence were acquired from a series of transversal sections of the Fast Blue-injected brain. The Fast Blue injection point on MPB that was identified from the serial sections (Figures 5A–D) was located in the ROI on MPB (Figures 5A,B). The injection point in the MPB was densely labeled by Fast Blue (Figure 6A, arrow). Fast Blue-labeled passing fibers were observed in the LPB and vsc (Figure 6B). However, there were no Fast Blue-labeled axons in scp and unc (Figure 6B). This observation was consistent with the labeling of lobule IX by AAV-CMV-hrGFP (Figure 2A).

**Figure 7:**
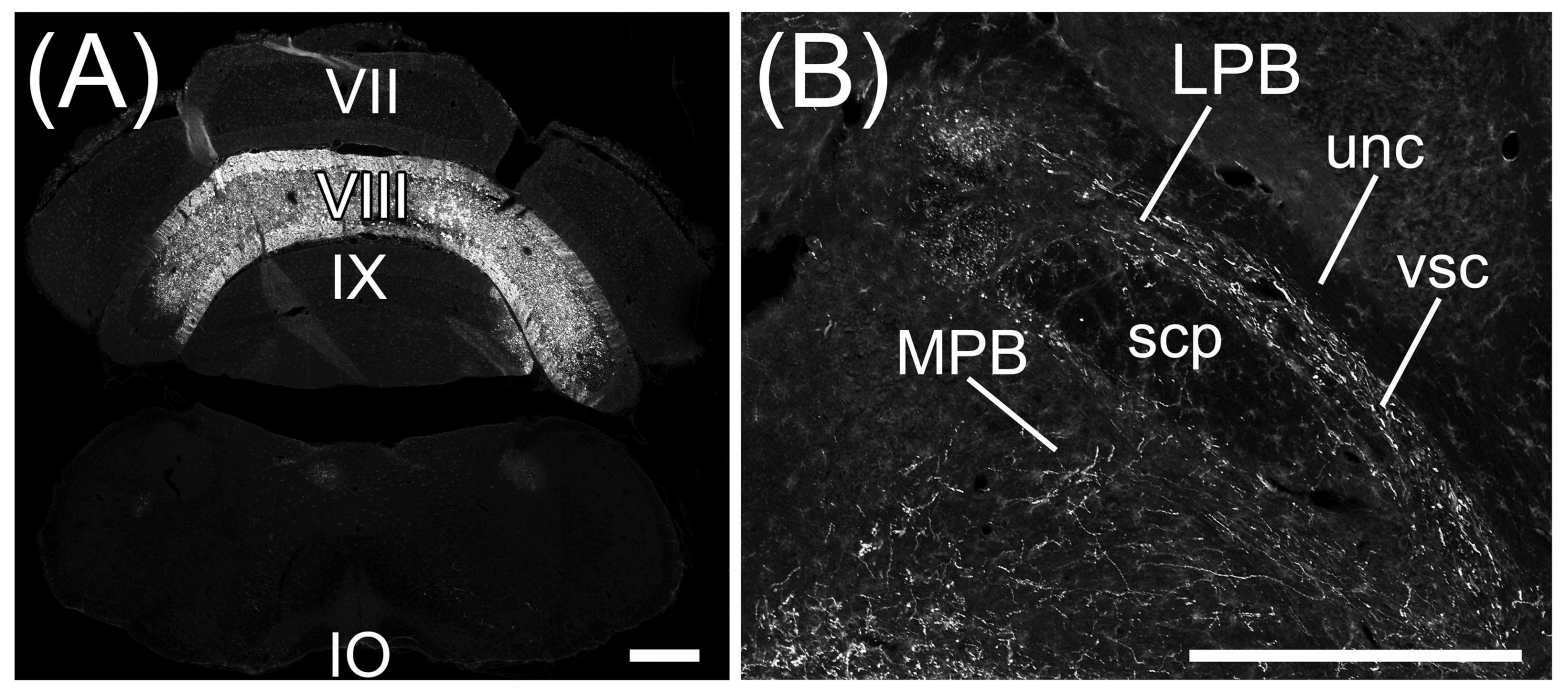
Projections to MPB from lobule VIII. Axons from cerebellar lobule VIII are anterogradely labeled by AAV-CMV-hrGFP. **(A)** Local injection of AAV-CMV-hrGFP to lobule VIII. Lobule VIII (VIII) is selectively and densely labeled by hrGFP. Inferior olive (IO) neurons are negative for hrGFP. **(B)** hrGFP-positive axons are abundant in LPB, MPB, and vsc, but not in scp and unc. VII, cerebellar lobule VII; IX, cerebellar lobule IX. Scale bar, 500 μm.

Interestingly, Fast Blue retrogradely labeled Purkinje cells in lobules VIII–X (Figures 6C',D',D”, arrowheads; lobule X, data not shown), FL (Figures 6E,E', arrowheads), and ventral PF (Figure 6E', arrowheads). Furthermore, Fast Blue-labeled Purkinje cells were observed only on the ipsilateral side of the Fast Blue-injected MPB (e.g., Figure 6C'). These results indicated that there are many Purkinje cells in lobules VIII–X, FL, and ventral PF that project their axons to the ipsilateral MPB. To confirm the axonal projection from cerebellar lobule VIII to the MPB, we locally injected AAV-CMV-hrGFP into cerebellar lobule VIII (*n* = 5, male; Figure 7A). hrGFP-positive axonal fibers were observed in the LPB, MPB, and vsc (Figure 7B). In addition, the scp and unc were negative for hrGFP (Figure 7B). This result (Figure 7B) was identical to the observation of AAV-CMV-hrGFP injection into lobule IX (Figures 1, 2A–C).

In contrast to lobules VIII–X, no Purkinje cells in lobules VI–VII were retrogradely labeled from the MPB by Fast Blue (Figure 6C', VII; Figure 6D”, VI,VII). This result indicated that Purkinje cells in lobules VI–VII had no projection to the MPB. This result was identical to the observation of AAV-CMV-hrGFP injection into lobule VI (Figures 2D–F).

In contrast to lobules VIII–X, very few Purkinje cells in lobules I–IV were retrogradely labeled from the MPB (Figure 6E”, arrowheads; lobule I, data not shown). Interestingly, these Fast Blue-labeled Purkinje cells were aligned like a band in the transverse section (Figure 6E”, arrowheads). In addition, Purkinje cells in the bilateral hemispheres were not labeled by Fast Blue (Figures 6D,E, arrows). Taken together, Purkinje cells in the vermis, mainly lobules VIII–X, directly project their axons to the MPB, and the vsc is the route of axonal connection between the cerebellar vermis and the MPB. In the cerebellar nuclei, Fast Blue retrogradely labeled the ipsilateral IntP but not the bilateral IntA and Lat from the MPB (Figure 6F). Furthermore, the ipsilateral Med, especially the ventral part of Med, was more labeled by Fast Blue than the contralateral Med to the Fast Blue-injected MPB (Figure 6F). This result indicated that the ipsilateral IntP and the ipsilateral Med projected their axons to the MPB.

IO and Pn were not labeled by Fast Blue-injection into the MPB (Figures 6C,D; Supplementary Figure 1G). The regions retrogradely labeled from MPB by Fast Blue were the dorsal part of medullary reticular nucleus (MdD; Figure 6C), the rostroventrolateral medulla (RVL; Figure 6C), Sol (Figures 6C,D), Sp5C (Figure 6C), the paratrigeminal nucleus (Pa5; Figure 6D), and the parvocellular reticular nucleus (PCR; Figures 6D,E). The other regions in the brain stem, midbrain, and cerebral cortex were also labeled by Fast Blue (Supplementary Figure 1). Almost all of the Fast Blue-labeled regions are consistent with previous papers indicating afferent connections of the MPB (Herkenham and Nauta, 1979; Cechetto et al., 1985; Moga et al., 1989; Herbert et al., 1990; Dietrichs et al., 1994; Caous et al., 2001; Vertes, 2004; Uschakov et al., 2007; Tokita et al., 2009; Coizet et al., 2010; Dobolyi et al., 2010; Nisimaru et al., 2013; Akhter et al., 2014).

### Retrograde labeling from medial vestibular nuclei by fast blue

**Figure 8:**
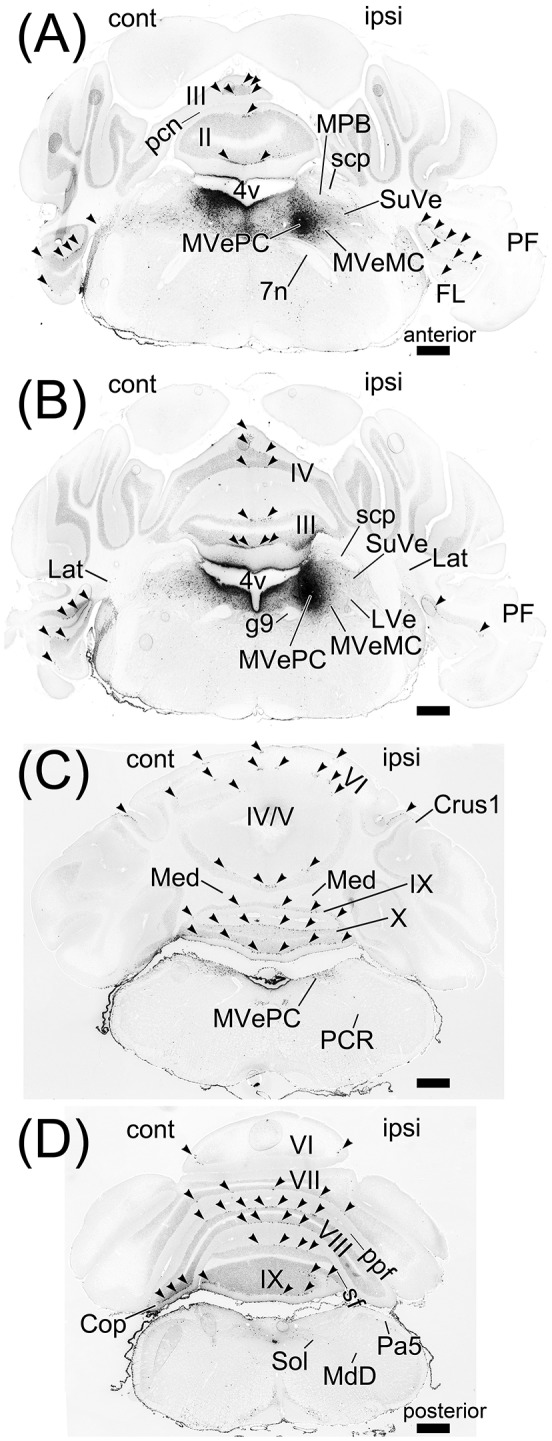
Projection neurons to medial vestibular nuclei. **(A–D)** Series of transversal sections from the anterior to posterior cerebellum. Fluorescence of Fast Blue on each section was imaged. The images are reversed to enhance visibility of Fast Blue labeling. Black dots are Fast Blue labeled neurons. **(A, B)** Fast Blue injection site. Fast Blue is locally injected into MVePC. The neurons projecting to MVePC are retrogradely labeled by Fast Blue. The arrowheads indicate the Fast Blue-positive Purkinje cells in lobules I–X, copula pyramidis (Cop), crus1 of ansiform lobule (Crus1), FL, and PF. The Fast Blue-positive Purkinje cells are observed on the bilateral side to the Fast Blue injected MVe. II–IX, numbers of cerebellar lobules; cont, contralateral; g7, genu of facial nucleus; ipsi, ipsilateral; LVe, lateral vestibular nucleus; MdD, dorsal part of medullary reticular nucleus; Pa5, paratrigeminal nucleus; pcn, precentral fissure; pcuf, preculminate fissure; ppf, prepyramidal fissure; sf, secondary fissure; SuVe, superior vestibular nucleus. Scale bar, 500 μm.

The vestibular nuclei are close to the MPB (Figure 2, lower panel; Figures 5C,G) and receive axonal inputs from cerebellar Purkinje cells (Thunnissen, 1990; Bukowska, 1995a,b). If the Fast Blue that was locally injected into the MPB had leaked into the neighboring vestibular nuclei, the presence of Purkinje cells labeled by Fast Blue might have indicated their projection from the cerebellar cortex to the vestibular nuclei in addition to their projection from the cerebellar cortex to the MPB. To confirm whether Fast Blue selectively labeled projection neurons to the MPB (Figure 6), we injected Fast Blue unilaterally into the medial vestibular nucleus (MVe; *n* = 4, male; Figures 5G,H, 8A,B) and compared the distribution of Fast Blue-positive neurons labeled from the MPB (Figure 6) and the distribution of Fast Blue-positive neurons labeled from the MVe (Figure 8). The Fast Blue injection point on MVe that was identified from the serial sections (Figures 5E–H) was located in MVePC (Figures 5G,H, 8A,B). The Fast Blue injection point on MVePC (Figures 5G,H) was posteriorly apart from the Fast Blue injection point on MPB (Figures 5A,B) and thus the Fast Blue injection points did not overlap each other. The Fast Blue-positive Purkinje cells were observed in cerebellar lobules I–X, the copula pyramidis (Cop), crus1 of the ansiform lobule (Crus1), FL, and PF and existed in the bilateral side of the cerebellum (Figure 8, arrowheads). By contrast, when Fast Blue was injected into the MPB, the Fast Blue-positive Purkinje cells were observed in lobules I–IV, lobules VIII–X, FL, and PF on the ipsilateral side of the Fast Blue injected MPB (Figure 6). These results indicated that the distribution of Purkinje cells that projected their axons to the MVe was different from the distribution of Purkinje cells that projected their axons to the MPB. If Fast Blue that was locally injected into the MPB had leaked into the MVe (Figure 6), Fast Blue would have labeled Purkinje cells in cerebellar lobule VI–VII. Furthermore, MdD, Pa5, PCR, and Sol were not retrogradely labeled from the MVe (Figures 8C,D), though they were retrogradely labeled from the MPB (Figures 6C–E). If Fast Blue that was locally injected into the MVe had leaked into the MPB, Fast Blue would have labeled MdD, Pa5, PRC, and Sol. Consequently, Fast Blue-leakage into the adjacent nuclei seems to be minimized.

### Retrograde labeling from MPB by AAV vector (retrograde serotype, rAAV2-retro)

**Figure 9:**
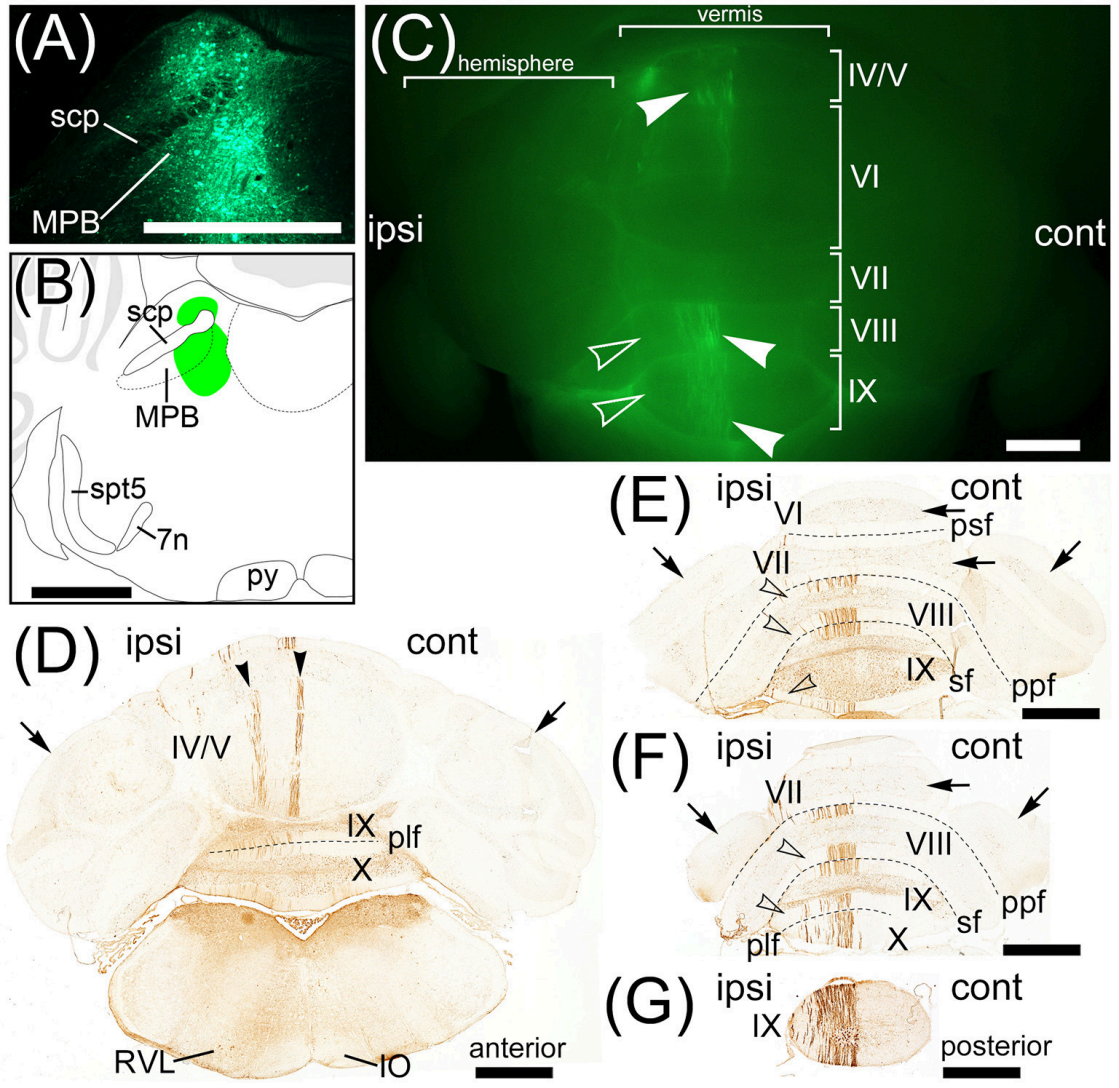
Projection neurons to MPB. Neurons projecting their axons to MPB were retrogradely labeled by AAV2retro-CAG-EGFP. **(A)** Local injection of AAV2retro-CAG-EGFP to MPB. The injection site is labeled by EGFP. **(B)** Schematic drawing of the AAV2retro-CAG-EGFP injection site. Green regions indicate AAV2retro-CAG-EGFP injected region. **(C)** Purkinje cells retrogradely labeled by AAV2retro-CAG-EGFP. The posterodorsal view of cerebellum is shown. Arrowheads indicate the EGFP-positive dendrites of Purkinje cells. The EGFP-positive dendrites are observed on the ipsilateral side (ipsi) but not on the contralateral side (cont) to the MPB injected with AAV2retro-CAG-EGFP. Unfilled arrowheads indicate the EGFP-negative lateral regions of lobules VIII and IX. **(D–G)** Series of transversal sections from posterior to anterior cerebellum immunostained with an anti-GFP antibody. The immunoreactivity to GFP is visualized by brown. Arrowheads indicate EGFP-positive bands in lobule IV/V. Arrows indicate the EGFP-negative hemispheres and lobules VI and VII. Unfilled arrowheads indicate the EGFP-negative lateral regions of lobules VIII–X. plf, posterolateral fissure; ppf, prepyramidal fissure; psf, posterior superior fissure; RVL, rostroventrolateral medulla; sf, secondary fissure. Scale bar, 1 mm.

A recent study reports an AAV vector performing efficiently retrograde gene-transfer, which is generated from AAV (serotype 2) by genetic modification of the viral capsid (Tervo et al., 2016). This AAV variant is named rAAV2-retro. A rAAV2-retro expressing a fluorescent protein is useful as a fluorescent retrograde tracer and the efficiency of retrograde labeling by rAAV2-retro is better than a classical retrograde tracer (Tervo et al., 2016). We generated AAV2retro-CAG-EGFP that is a rAAV2-retro expressing EGFP under the control of CAG promoter. To strengthen the results of retrograde labeling from MPB by Fast Blue, we locally injected AAV2retro-CAG-EGFP into the unilateral MPB (*n* = 6, male; Figure 9). One week after the injection, the injection point in the MPB was densely labeled by EGFP (Figure 9A) and located in the ROI on MPB (Figure 9B). The AAV2retro-CAG-EGFP was efficiently transported from axonal terminals to cell bodies of projection neurons of MPB and expressed EGFP in the projection neurons. As a result, cell bodies, dendrites, and axons of the projection neurons were visualized by the EGFP filling the projection neurons. EGFP-positive signals were observed in the cerebellum (Figure 9C). The EGFP-positive signals were located in the ipsilateral side to the MPB injected with AAV2retro-CAG-EGFP and observed in the lobule IV/V, lobule VIII, and lobule IX of the cerebellar vermis (Figure 9C, arrowheads). The lobule VI, lobule VII, and hemisphere were negative for EGFP. The serial transverse sections of the cerebellum were immunostained with anti-GFP antibody to enhance the EGFP signals (Figure 9D–G). The EGFP-positive signals in the cerebellum (Figure 9C, arrowheads) were identified as the dendrites of Purkinje cells labeled by EGFP. The EGFP-positive Purkinje cells were located in the ipsilateral side to the MPB injected with AAV2retro-CAG-EGFP (Figures 9D–G). In the lobules IV/V, the EGFP-positive Purkinje cells were aligned like a band and formed EGFP-positive two narrow bands (Figure 9D, arrowheads). In the lobules VIII–X, the EGFP-positive Purkinje cells were preferentially located in the medial region of lobules VIII–X and formed one wide band (Figure 9C, arrowheads in the lobules VIII and IX; Figures 9E–G). The lateral region of lobules VIII–X were negative for EGFP (Figure 9C, unfilled arrowheads; Figures 9E,F, unfilled arrowheads). Purkinje cells in the lobule VI, lobule VII, FL, PF, and hemispheres were negative for EGFP (Figures 9D–F, arrows; FL and PF, data not shown). The distribution of EGFP-positive Purkinje cells (Figures 9D–G) was similar to the distribution of Purkinje cells labeled by Fast Blue from MPB except for FL and PF (Figure 6). Pa5, PCR, RVL, Sol, and Sp5C were retrogradely labeled from MPB by AAV2retro-CAG-EGFP (Figure 9D; Supplementary Figure 2G) in the same way as the Fast Blue labeling from MPB (Figures 6C–E). The EGFP-positive regions in the brain stem, midbrain, and cerebellar cortex (Supplementary Figure 2) were identical with the brain regions labeled from MPB by Fast Blue (Supplementary Figure 1).

### Retrograde labeling from lobule IX by fast blue

**Figure 10:**
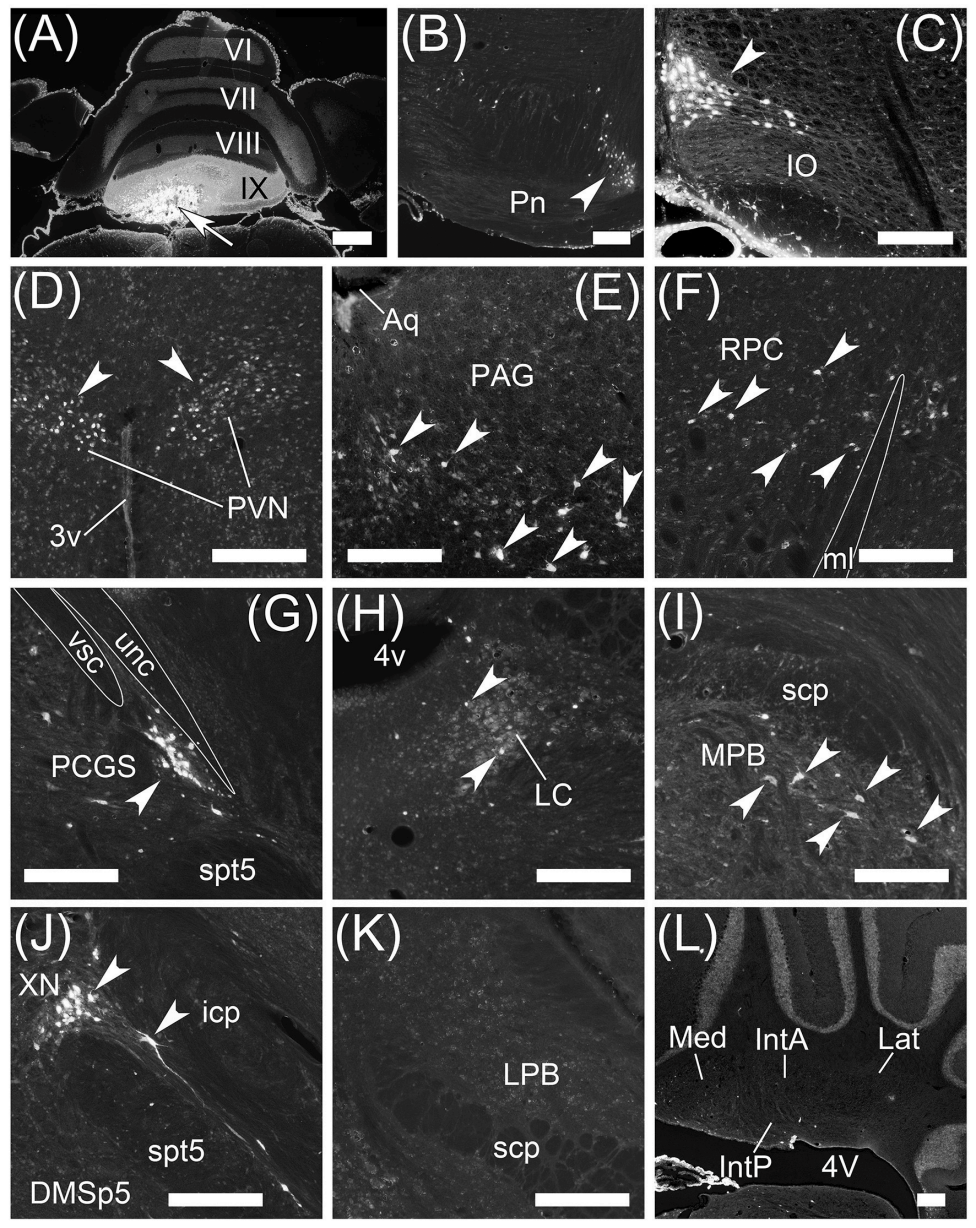
Projection neurons to cerebellar lobule IX. Neurons projecting to cerebellar lobule IX were retrogradely labeled by Fast Blue. **(A)** Local injection of Fast Blue to cerebellar lobule IX. The injection site is indicated by an arrow. **(B–J)** Retrogradely labeled neurons from cerebellar lobule IX in dorsolateral Pn (**B**, arrowhead), beta subnucleus of IO (**C**, arrowhead), paraventricular hypothalamic nucleus (PVN; **D**, arrowheads), periaqueductal gray (PAG; **E**, arrowheads), parvocellular part of red nucleus (RPC; **F**, arrowheads), PCGS (**G**, arrowhead), LC (**H**, arrowheads), MPB (**I**, arrowheads), and XN (**J**, arrowheads). **(F)** White line indicates the shape of medial lemniscus (ml). **(G)** White lines indicate the shape of unc and vsc. **(K)** Fast Blue-negative LPB. LPB neurons are not retrogradely labeled from lobule IX by Fast Blue. **(L)** Fast Blue-negative cerebellar nuclei. The cerebellar nuclei (IntA, IntP, Lat, and Med) were not labeled by Fast Blue. The left side in **(B**,**C**,**E–L)** is the medial side of brain section. 3v, third ventricle; 4v, fourth ventricle; Aq, aqueduct; DMSp5, dorsomedial spinal trigeminal nucleus; spt5, spinal trigeminal tract. Scale bar, 500 μm in **(A)**, 200 μm in **(B–L)**.

To examine brain regions projecting to lobule IX, we locally injected Fast Blue into lobule IX (*n* = 5, male; Figure 10). Lobule IX was selectively labeled by Fast Blue (Figure 10A). The other lobules, FL, PF, and hemisphere were not labeled by Fast Blue (Figure 10A; data not shown). The dorsolateral region of Pn (Figure 10B, arrowhead) and beta subnucleus of IO (Figure 10C, arrowhead) were retrogradely labeled from lobule IX by Fast Blue. Previous reports indicate that these subnuclei of Pn and IO project their axons to lobule IX, respectively (Bernard, 1987; Sugihara and Shinoda, 2004, 2007; Voogd and Ruigrok, 2004; Ruigrok et al., 2015). This indicates that the retrograde labeling from lobule IX was performed successfully. The paraventricular hypothalamic nucleus (PVN; Figure 10D, arrowheads), ventrolateral region of periaqueductal gray (PAG; Figure 10E, arrowheads), parvocellular part of red nucleus (RPC; Figure 10F, arrowheads), paracochlear glial substance (PCGS) (which is a part of the cochlear nuclei) (Figure 10G, arrowhead), LC (Figure 10H, arrowheads), MPB (Figure 10I, arrowheads), which was within the ROI, and XN (which is a part of vestibular nuclei) (Figure 10J, arrowheads) projected to cerebellar lobule IX. Notably, PAG and PVN projected their axons to both lobule IX (Figures 10D,E) and the MPB (Supplemental Figures 1C,F,G). By contrast, lobule IX did not project to the LC (Figures 3A–C), PAG (data not shown), PCGS (Figure 1I'), PVN (data not shown), RPC (data not shown), and XN (Figures 1E'–G'). The LPB and cerebellar nuclei (IntA, IntP, Lat, and Med) did not project to lobule IX (LPB, Figure 10K; the cerebellar nuclei, Figure 10L). The cerebellar nuclei are close to lobule IX (Figures 1B',C'). If Fast Blue that was locally injected into lobule IX had leaked into the neighboring cerebellar nuclei, Fast Blue would have labeled the cerebellar nuclei. In fact, the cerebellar nuclei were negative for Fast Blue (Figure 10L). Therefore, these results indicated that Fast Blue was locally injected into lobule IX without leaking into the neighboring cerebellar nuclei. The neural circuits between cerebellar lobule IX and the MPB are summarized in Figure 12.

**Figure 11:**
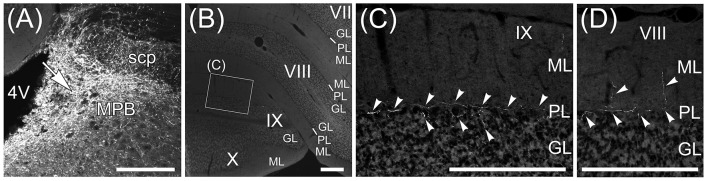
Beaded fibers from MPB. Axons from MPB are anterogradely labeled by AAV-CMV-hrGFP. **(A)** Local injection of AAV-CMV-hrGFP to MPB. An arrow indicates the injection site. MPB is densely labeled by hrGFP. **(B)** The transversal section of cerebellum. The cerebellar lobules VII–X are indicated. **(C)** hrGFP-positive beaded fiber in lobule IX. The magnified image of the quadrilateral region in **(B)** is indicated. Arrowheads indicate the hrGFP-positive beaded fiber, which is along the Purkinje cell layer (PL). **(D)** hrGFP-positive beaded fiber in lobule VIII. The cerebellar section different from **(B)** is indicated. The arrowheads indicate the hrGFP-positive beaded fibers extending from PL to ML. 4V, fourth ventricle; GL, granular layer; ML, molecular layer. Scale bar, 200 μm.

**Figure 12:**
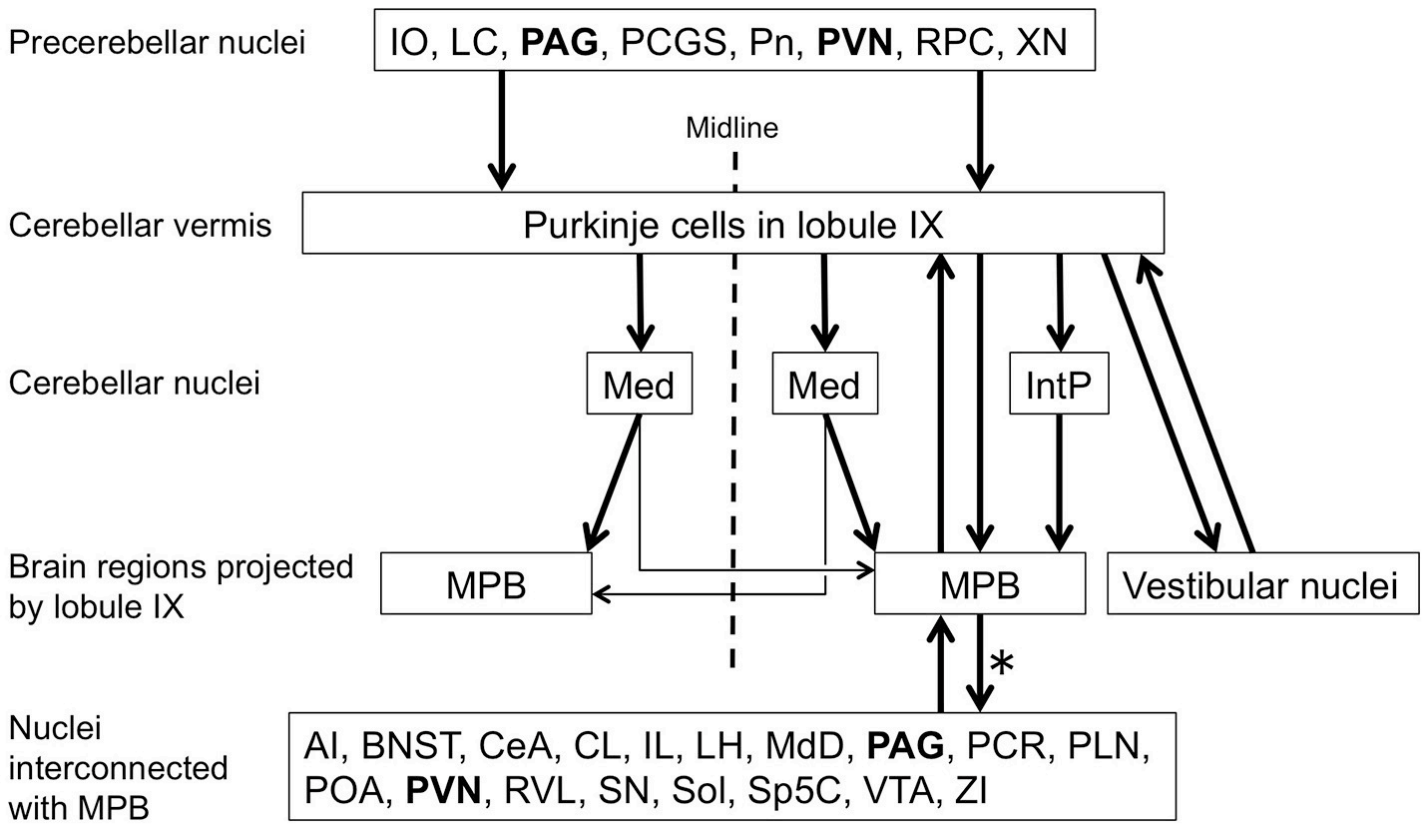
Putative circuit associated with lobule IX of cerebellar vermis and MPB. Precerebellar nuclei of lobule IX, cerebellar nuclei projected by lobule IX, and brain regions projected by lobule IX are indicated according our results. Purkinje cells in lobule IX project to the ipsilateral IntP and Med. IntP projects to the ipsilateral MPB but Med mainly projects to the contralateral MPB. Lobule IX also directly projects to the ipsilateral MPB and vestibular nuclei. MPB interconnect with many brain regions, including ipsilateral lobule IX of the cerebellar vermis. Efferent projections of MPB (asterisk) refer to previous reports (Saper and Loewy, 1980; Fulwiler and Saper, 1984; Bernard et al., 1993; Feil and Herbert, 1995; Chamberlin et al., 1998; Len and Chan, 1999). Periaqueductal gray (PAG) and Paraventricular hypothalamic nucleus (PVN) get inputs from MPB and project to lobule IX. Therefore, a loop circuit is formed among PAG/PVN, lobule IX, and MPB. AI, Agranular insular cortex; BNST, Bed nucleus of the stria terminalis lateral division; CeA, Central amygdaloid nuclei; CL, Claustrum; IL, Infralimbic cortex; IO, inferior olive; LC, locus ceruleus; LH, Lateral hypothalamic area; MdD, dorsal part of medullary reticular nucleus; PCGS, paracochlear glial substance; PLN, Paralemniscal nucleus; Pn, Pontin nucleus; POA, Preoptic area; RPC, Parvocellular part of red nucleus; RVL, rostroventrolateral medulla; SN, Substantia nigra; VTA, Ventral tegmental area; XN, X nucleus; ZI, Zona incerta.

### Anterograde labeling from MPB by AAV-CMV-hrGFP

The Fast Blue-labeling of lobule IX indicated that MPB neurons projected to lobule IX (Figure 10I). To examine axonal inputs from the MPB to the cerebellum, we locally injected AAV-CMV-hrGFP into the unilateral ROI on MPB (*n* = 4, male; Figure 11). Fluorescence of hrGFP on a series of transversal sections of the AAV-CMV-hrGFP-injected brain was imaged. The MPB was densely labeled by hrGFP at the injection site of AAV-CMV-hrGFP Figure 11A, arrow). In contrast to the Fast Blue-injection into the MPB (Figure 6), there were no hrGFP-positive cells in the cerebellar cortex (Figures 11B–D). The cerebellum receives three distinct types of axonal inputs; mossy fibers existing in the granular layer (GL), climbing fibers existing in the molecular layer (ML), and beaded fibers along the Purkinje cell layer (PL) (King et al., 1992). The mossy and climbing fibers in cerebellar lobules VII–X were negative for hrGFP (Figures 11B–D). By contrast, there were many hrGFP-positive beaded fibers in lobules VIII–X. hrGFP-positive beaded fibers were observed along the Purkinje cell layer (PL; Figure 11C, arrowheads), and a part of the hrGFP-positive beaded fibers extended to the molecular layer (ML; Figure 11D, arrowheads).

The vestibular nuclei that are close to the MPB (Figure 2, lower panel; Figures 5C,G) project their axons to the cerebellum as mossy fibers (Thunnissen, 1990). If AAV-CMV-hrGFP that was locally injected into the MPB had leaked into the neighboring vestibular nuclei, hrGFP-positive mossy fibers would have been observed in the cerebellum. However, hrGFP-positive mossy fibers were not observed (Figures 11B–D). Therefore, local injection into the MPB was successful without unexpected labeling of the neighboring vestibular nuclei.

## Discussion

Using anterograde labeling with an AAV vector, AAV-CMV-hrGFP (serotype rh10), and retrograde labeling by Fast Blue and an AAV vector, AAV2retro-CAG-EGFP (retrograde serotype), we found a direct connection between lobule IX of the mouse cerebellar vermis and the MPB. In contrast to lobule IX, there is no axonal connection between the lobule VI of the cerebellar vermis and the MPB. The direct connection between lobule IX and the MPB is a special neuronal circuit in the cerebellum, and this suggests that the cerebellum participates in regulating the sleep-wake cycle and the cardiovascular and respiratory responses by modulating the neural activity of the MPB.

### Anterograde labeling with AAV vector serotype rh10

Recently, viral vectors expressing a fluorescent protein, such as GFP, have been applied to neural circuit labeling (e.g., AAV vector, adenoviral vector, Herpes Simplex viral vector, and Rabies viral vector). Each viral vector has a different viral tropism and efficiency of gene transfer (e.g., AAV vectors; Aschauer et al., 2013; Murlidharan et al., 2014). Viral vectors usually infect cell bodies of neurons at the viral vector injection site and perform anterograde labeling of the axons and dendrites by a fluorescent protein. By contrast, some viral vectors (e.g., adenoviral vector, Bru et al., 2010; Hashimoto et al., 2012; AAV vector, Tervo et al., 2016; Rabies viral vector, Wickersham et al., 2007) infect axonal terminals at the viral vector injection site and are retrogradely transported from the axonal terminal to the cell body of projection neuron, and as a result, the projection neurons are labeled by a fluorescent protein. If we use a viral vector for neural circuit labeling, it is necessary to select a viral vector that is suitable for the target brain region and for anterograde or retrograde neuronal circuit labeling. Our observations indicated that an AAV vector (serotype rh10) showed highly efficient gene transfer to lobule IX of the cerebellar vermis without retrograde labeling of projection neurons to lobule IX (Figure 1), in contrast to the other serotypes of AAV vectors (Murlidharan et al., 2014). Furthermore, the AAV vector has an advantage over classical anterograde tracers of *Phaseolus vulgaris* leucoagglutinin (PHA-L) and biotinylated dextran amine (BDA) because the AAV vector is not incorporated by the passing fibers at the AAV vector injection site, unlike PHA-L and BDA (Chamberlin et al., 1998; Wang et al., 2014). Therefore, AAV-CMV-hrGFP, which is an AAV vector (serotype rh10) expressing hrGFP, was suitable for anterograde labeling of the efferent fibers of the local region of the cerebellum.

### Retrograde labeling with fast blue

When Fast Blue is injected into MPB as a retrograde neuronal tracer, Fast Blue-labeling via axonal fibers passing through a Fast Blue-injection site and Fast Blue-leakage into the nuclei adjacent to MPB, which are LC, LVe, MVe, and SuVe, become subjects of discussion. Fast Blue is more readily taken up via intact axonal terminals than the passing fibers (Köbbert et al., 2000). Therefore, it is considered that Fast Blue hardly labels axonal fibers passing through a Fast Blue-injection site. Fast Blue-leakage into the adjacent nuclei of MPB was evaluated by the comparison between Fast Blue-labeling from MPB (Figure 6) and Fast Blue-labeling from MVePC (Figure 8). Fast Blue is locally injected into the MPB (Figures 5A,B) and the MVePC (Figures 5G,H). The Fast Blue injection region on the MPB does not overlap with the Fast Blue injection region on the MVePC (Figure 5). The distribution of Fast Blue-labeled neurons projecting to MPB was different from the distribution of Fast Blue-labeled neurons projecting to MVePC. Consequently, we concluded that Fast Blue-leakage into the adjacent nuclei of MPB was minimized. However, further investigations on the afferent inputs to MPB are needed because it is difficult to completely eliminate Fast Blue-leakage into the nuclei adjacent to MPB. Our observation indicated that cerebellar lobule IX did not project to LC (Figure 3A) but Schwarz et al. (2015) indicate direct connection from cerebellar Purkinje cells in lobule IX to LC by rabies-virus-mediated retrograde neuronal tracing. This contradiction needs to be investigated in the future. The distribution of MPB-projecting neurons labeled by Fast Blue (Figure 6; Supplementary Figure 1) is similar to that of MPB-projecting neurons labeled by AAV2retro-CAG-EGFP (Figure 9; Supplementary Figure 2). This result strengthens the reliability of Fast Blue labeling from MPB. However, Fast Blue labels the FL and the ventral PF from MPB, but AAV2retro-CAG-EGFP does not. This discrepancy between Fast Blue-labeling and AAV2retro-CAG-EGFP labeling should be examined by an anterograde-labeling from FL and PF in the future.

### Local heterogeneity of the connection from cerebellum to MPB

Retrograde labeling from the MPB indicated that the ipsilateral Purkinje cells in the cerebellar vermis directly projected to the MPB (Figures 6, 9). Furthermore, the direct connection between Purkinje cells of the cerebellar vermis and MPB is locally Non-uniform (Figures 6C',D',D”,E”, 9C–G). Many Purkinje cells in lobules VIII–X directly project to the ipsilateral MPB, but there is no axonal connection from Purkinje cells in lobules VI–VII to the MPB (Figures 6C',D”, 9C–G). In contrast to lobules VIII–X, a small population of Purkinje cells in lobules I–V project to the ipsilateral MPB and they are aligned and form a sagittal band in lobules I–V (Figure 6E”, arrowheads; Figure 9D, arrowheads). The sagittal band in lobules I–V seems to concur with the zebrine II/aldolase C-positive sagittal band 2+ that is identical to zone Ax, which is formed by terminals of climbing fibers that originate from specific subnuclei of the inferior olive (Sugihara and Shinoda, 2004, 2007; Voogd and Ruigrok, 2004; Namba et al., 2011; Fujita et al., 2014). The local heterogeneity of the axonal connection from the cerebellar vermis to the MPB suggests functional differences among lobules I–V, lobules VI–VII, and lobules VIII–X of the cerebellar vermis. Lobules VIII–X of the cerebellar vermis seem to be at the center of regulating the functions of the MPB from the viewpoint of the dense neural connection between lobules VIII–X and the MPB.

### Cerebellar neural circuit contributing to the sleep-wake cycle, and cardiovascular and respiratory responses

The direct connection between lobules VIII–X and the MPB suggests that lobules VIII–X regulate the neural activity of the MPB. The MPB is involved in switching from REM sleep to Non-REM sleep and *vice versa* (Fuller et al., 2011; Anaclet et al., 2012; Hayashi et al., 2015), and cardiovascular and respiratory responses (Nisimaru, 2004; Song et al., 2006). The neural circuits associated with cerebellar lobule IX and the MPB are summarized in Figure 12 in accordance with our observations and previous reports (Saper and Loewy, 1980; Fulwiler and Saper, 1984; Bernard et al., 1993; Feil and Herbert, 1995; Chamberlin et al., 1998). The LC, PAG, and PVN project their axons to lobule IX of the cerebellar vermis as precerebellar nuclei. The LC and PVN are involved in regulating the sleep-wake cycle (for a review see Tsujino and Sakurai, 2013). The ventrolateral part of the PAG contributes to negative regulation of REM sleep (Lu et al., 2006; Sapin et al., 2009). Lobule IX directly projects to the ipsilateral MPB. The MPB interconnects with many brain regions, including ipsilateral lobule IX of the cerebellar vermis (refer to Figure 12, Nuclei interconnected with MPB). The BNST, CeA, IL, LH, PAG, POA, PVN, Sol, VTA, and ZI of the nuclei interconnected with the MPB participate in regulating the sleep-wake cycle (for reviews see de Andrés et al., 2011; Tsujino and Sakurai, 2013). Furthermore, the LH, PAG, PVN, RVL, and Sol of the nuclei interconnected with the MPB participate in regulating the cardiovascular and respiratory responses (for reviews see Nisimaru, 2004; Burdakov et al., 2013). Especially, RVL is the center of cardiovascular control. Interestingly, PAG and PVN form a feedback loop with lobule IX through the MPB (Figure 12). This loop circuit seems to be important for the physiological function of lobule IX regulating the sleep-wake cycle, and cardiovascular and respiratory responses. It is supported by the fact that MPB, PAG, and PVN are involved in the cardiovascular control during sleep (for a review see Silvani and Dampney, 2013). Patients with cerebellar malformation (e.g., spinocerebellar ataxia) often suffer from sleep disturbances, especially sleep apnea that is characterized by repeated pauses in breathing during sleep (Pedroso et al., 2011; DelRosso and Hoque, 2014; Canto et al., 2017). This supports that the cerebellum contributes to breathing during sleep. Taking all the above facts into account, lobule IX of the cerebellar vermis appears to participate in the neural circuit of regulating the sleep-wake cycle, and cardiovascular and respiratory responses through the MPB. Lobules VIII and X are thought to establish the same neural circuit as lobule IX; however, this has not been confirmed. It is necessary to examine the neural circuit in which lobule VIII and lobule X participate. Furthermore, we need to examine the physiological connectivity between the cerebellum and the MPB with electrophysiological techniques, and determine whether cerebellar activity modulates the physiological function of the MPB that is involved in regulating the sleep-wake cycle, and cardiovascular and respiratory responses. The direct axonal connection between cerebellar lobules VIII–X and the MPB will help us to elucidate the relationship between cerebellar activity and physiological function regulating the sleep-wake cycle and the cardiovascular and respiratory responses.

## Author contributions

MH designed the study, analysis and interpretation of data, and wrote the initial draft of the manuscript. AY constructed and purified AAV-CMV-hrGFP (serotype rh10). MT constructed AAV vector (serotype 9). SK and KK constructed and purified AAV2retro-CAG-EGFP. HY contributed to data interpretation and critically reviewed the manuscript. The final version of the manuscript was approved by all authors.

### Conflict of interest statement

The authors declare that the research was conducted in the absence of any commercial or financial relationships that could be construed as a potential conflict of interest.

## Supplementary material

Supplementary Figure 1 Distribution of neurons labeled retrogradely from MPB by Fast Blue. **(A–G)** Series of transversal sections from posterior to anterior brain. The images are reversed to increase visibility of sections. Therefore, black dots are neurons that are retrogradely labeled from MPB by Fast Blue. AI, agranular insular cortex; BNST, bed nucleus of the stria terminalis lateral division; CeA, central amygdaloid nuclei; CL, clausrum; CPu, caudate putamen; DP, dorsal peduncular cortex; IL, infralimbic cortex; LH, lateral hypothalamic area; LHB, lateral habenular nucleus; POA, preoptic area; PAG, periaqueductal gray; PLN, paralemniscal nucleus; Pn, Pontin nucleus; PVN, paraventricular hypothalamic nucleus; SN, substantia nigra; VMH, ventromedial hypothalamic nucleus; VTA, ventral tegmental area; ZI, zona incerta. Scale bar, 1 mm.

Supplementary Figure 2 . Distribution of neurons labeled retrogradely from MPB by AAV2retro-CAG-EGFP. **(A–G)** Series of transversal sections from posterior to anterior brain. Fluorescence of EGFP on each section was imaged. The images are converted to black and white and reversed to enhance visibility of sections. Therefore, black neurons and fibers are EGFP-positive. fr, fasciculus retroflexus; Scale bar, 500 μm.

Supplementary Movie 1 3D-reconstruction from the confocal image of MPB immunostained with anti-SYP antibody. The movie of the 3D image is reconstructed from the confocal image of Figure 4I. The hrGFP-fluorescence is green-colored. Immunostaining with anti-SYP antibody is red-colored. Arrows indicate the axonal terminals that are double-positive for hrGFP and SYP (yellow color).

Supplementary Movie 2 3D-reconstruction from the confocal image of MPB immunostained with anti-VGLUT2 antibody. The movie of the 3D image is reconstructed from the confocal image of Figure 4L. The hrGFP-fluorescence is green-colored. Immunostaining with anti-VGLUT2 antibody is red-colored. Arrowheads indicate the VGLUT2-positive MPB cells that are surrounded by the hrGFP-positive axonal terminals.

Supplementary Movie 3 3D-reconstruction from the confocal image of MPB immunostained with anti-CGRP antibody. The movie of the 3D image is reconstructed from the confocal image of Figure 4O. The hrGFP-fluorescence is green-colored. Immunostaining with anti-CGRP antibody is red-colored. Arrowheads indicate the CGRP-positive MPB cells that are surrounded by the hrGFP-positive axonal terminals.

## Abbreviations

3v: third ventricle
4v: fourth ventricle
7n: root of facial nucleus
Aq: aqueduct
cbc: cerebellar commissure
CGRP: calcitonin gene-related peptide
cont: contralateral
Cop: copula pyramidis
Crus1: crus1 of ansiform lobule
Cu: cuneate nucleus
DMSp5: dorsomedial spinal trigeminal nucleus
DTg: dorsal tegmental nucleus
ECu: external cuneate nucleus
FL: flocculus
g7: genu of facial nucleus
Gi: gigantocellular reticular nucleus
GL: granular layers
hrGFP: humanized renilla green fluorescent protein
icp: inferior cerebellar peduncle
ipsi: ipsilateral
IntA: anterior interposed cerebellar nucleus
IntP: posterior interposed cerebellar nucleus
IO: inferior olive
Lat: lateral (dentate) cerebellar nucleus
LC: locus coeruleus
LPB: lateral parabrachial nucleus
LVe: lateral vestibular nucleus
LRt: lateral reticular nucleus
mcp: medial cerebellar peduncle
MdD: dorsal part of medullary reticular nucleus
MdV: ventral part of medullary reticular nucleus
Med: medial (fastigial) cerebellar nucleus
ML: molecular layer
Mo5: motor trigeminal nucleus
MPB: medial parabrachial nucleus
mscp: the medial-most region of superior cerebellar peduncle
MVe: medial vestibular nucleus
MVeMC: magnocellular part of medial vestibular nucleus
MVePC: parvocellular part of medial vestibular nucleus
Pa5: paratrigeminal nucleus
PAG: ventrolateral region of periaqueductal gray
PCGS: paracochlear glial substance
pcn: precentral fissure
pcuf: preculminate fissure
PCR: parvocellular reticular nucleus
PF: paraflocculus
plf: posterolateral fissure
Pn: pontine nucleus
PVN: paraventricular hypothalamic nucleus
ppf: prepyramidal fissure
Pr: prepositus nucleus
py: pyramidal tract
Pr5: principal sensory trigeminal nucleus
psf: posterior superior fissure
RPC: parvocellular part of red nucleus
RVL: rostroventrolateral medulla
s5: sensory root of trigeminal nucleus
scp: superior cerebellar peduncle
sf: secondary fissure
Sol: nucleus of the solitary tract
Sp5C: caudal part of spinal trigeminal nucleus
SP5I: interpolar part of spinal trigeminal nucleus
spt5: spinal trigeminal tract
SpVe: spinal vestibular nucleus
Su5: supratrigeminal nucleus
SuVe: superior vestibular nucleus
SYP: synaptophysin
TH: tyrosine hydroxylase
unc: uncinate fasciculus of cerebellum
VGLUT2: vesicular glutamate transporter 2
vsc: ventral spinocerebellar tract
XN: X nucleus.
