# Supplementary figures and images for: Anatomical Evidence for a Direct Projection from Purkinje Cells in the Mouse Cerebellar Vermis to Medial Parabrachial Nucleus
Source: Front Neural Circuits. 2018 Feb 7;12:6. doi: 10.3389/fncir.2018.00006 (PMC5808303; doi:10.3389/fncir.2018.00006)

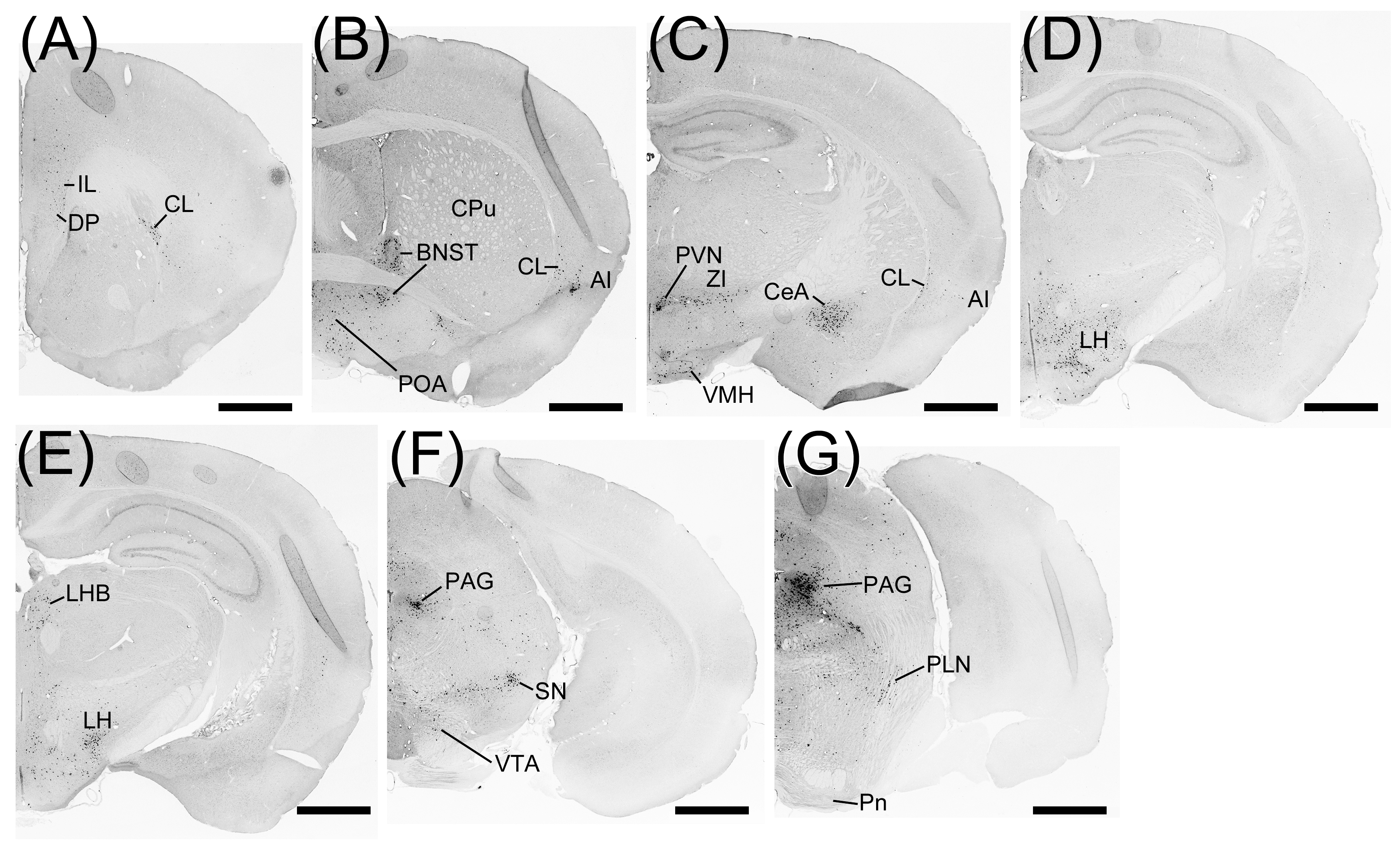

Supplement: Supplementary Figure 1 — Distribution of neurons labeled retrogradely from MPB by Fast Blue. (A–G) Series of transversal sections from posterior to anterior brain. The images are reversed to increase visibility of sections. Therefore, black dots are neurons that are retrogradely labeled from MPB by Fast Blue. AI, agranular insular cortex; BNST, bed nucleus of the stria terminalis lateral division; CeA, central amygdaloid nuclei; CL, clausrum; CPu, caudate putamen; DP, dorsal peduncular cortex; IL, infralimbic cortex; LH, lateral hypothalamic area; LHB, lateral habenular nucleus; POA, preoptic area; PAG, periaqueductal gray; PLN, paralemniscal nucleus; Pn, Pontin nucleus; PVN, paraventricular hypothalamic nucleus; SN, substantia nigra; VMH, ventromedial hypothalamic nucleus; VTA, ventral tegmental area; ZI, zona incerta. Scale bar, 1 mm. [file Image1.JPEG]

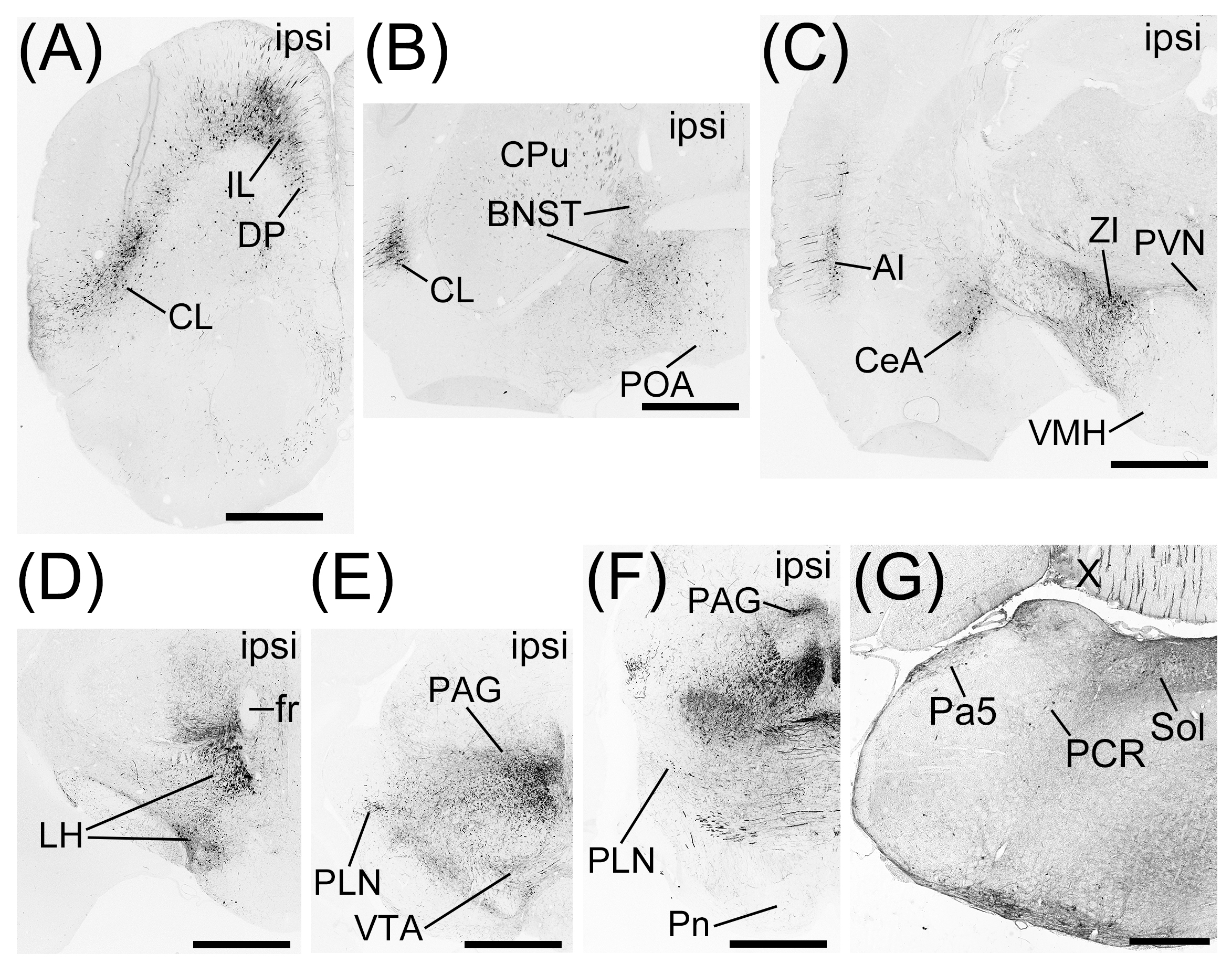

Supplement: Supplementary Figure 2 — . Distribution of neurons labeled retrogradely from MPB by AAV2retro-CAG-EGFP. (A–G) Series of transversal sections from posterior to anterior brain. Fluorescence of EGFP on each section was imaged. The images are converted to black and white and reversed to enhance visibility of sections. Therefore, black neurons and fibers are EGFP-positive. fr, fasciculus retroflexus; Scale bar, 500 μm. [file Image2.JPEG]
